# Gene Expression Profiling of Colorectal Tumors and Normal Mucosa by Microarrays Meta-Analysis Using Prediction Analysis of Microarray, Artificial Neural Network, Classification, and Regression Trees

**DOI:** 10.1155/2014/634123

**Published:** 2014-05-19

**Authors:** Chi-Ming Chu, Chung-Tay Yao, Yu-Tien Chang, Hsiu-Ling Chou, Yu-Ching Chou, Kang-Hua Chen, Harn-Jing Terng, Chi-Shuan Huang, Chia-Cheng Lee, Sui-Lun Su, Yao-Chi Liu, Fu-Gong Lin, Thomas Wetter, Chi-Wen Chang

**Affiliations:** ^1^Division of Bioinformatics and Statistics, School of Public Health, National Defense Medical Center, Taipei 114, Taiwan; ^2^Department of Surgery, Cathay General Hospital, Taipei 106, Taiwan; ^3^Department of Nursing, Oriental Institute of Technology and Far Eastern Memorial Hospital, New Taipei City 220, Taiwan; ^4^Department of Epidemiology, School of Public Health, National Defense Medical Center, Taipei 114, Taiwan; ^5^Department of Nursing, College of Medicine, Chang Gung University, Taoyuan 333, Taiwan; ^6^Advpharma, Inc., New Taipei City 221, Taiwan; ^7^Division of Colorectal Surgery, Cheng Hsin Rehabilitation Medical Center, Taipei 112, Taiwan; ^8^Division of Colon and Rectal Surgery, Department of Surgery, Tri-Service General Hospital, Taipei 114, Taiwan; ^9^Division of Surgery, Tri-Service General Hospital, Taipei 114, Taiwan; ^10^Department of Medical Informatics, Faculty of Medicine, University of Heidelberg, Heidelberg 69120, Germany

## Abstract

*Background*. Microarray technology shows great potential but previous studies were limited by small number of samples in the colorectal cancer (CRC) research. The aims of this study are to investigate gene expression profile of CRCs by pooling cDNA microarrays using PAM, ANN, and decision trees (CART and C5.0). *Methods*. Pooled 16 datasets contained 88 normal mucosal tissues and 1186 CRCs. PAM was performed to identify significant expressed genes in CRCs and models of PAM, ANN, CART, and C5.0 were constructed for screening candidate genes via ranking gene order of significances. *Results*. The first screening identified 55 genes. The test accuracy of each model was over 0.97 averagely. Less than eight genes achieve excellent classification accuracy. Combining the results of four models, we found the top eight differential genes in CRCs; suppressor genes, *CA7, SPIB, GUCA2B, AQP8, IL6R* and *CWH43;* oncogenes, *SPP1* and *TCN1*. Genes of higher significances showed lower variation in rank ordering by different methods. *Conclusion*. We adopted a two-tier genetic screen, which not only reduced the number of candidate genes but also yielded good accuracy (nearly 100%). This method can be applied to future studies. Among the top eight genes, *CA7*, *TCN1*, and *CWH43* have not been reported to be related to CRC.

## 1. Background

Colorectal cancer is one of the leading cancers in the world and considered to be among the most frequent causes of death, along with lung, prostate, and breast cancer [1]. Microarray analysis has provided insights into the genomic alterations that potentially underpin the processes of colorectal carcinogenesis, tumor growth, and metastasis and has enabled the identification of gene signatures for diagnosis, molecular characterization, prognosis prediction, and treatment prediction [2]. However, there remains a lack of clinically useful biomarkers emerging for cancers [3]. The translation of microarrays analysis into clinical practice is still far from complete for several reasons: (1) the lack of comparison and overlap of results obtained from each individual study [4–6] due to technique-related variability of sample collections and preparation, type of platform used, and data analysis; (2) the lack of large-scale studies due to the small number of available patient samples, leading to reduced statistical power [7]; (3) the difficulty in understanding and selecting the data that would be informative and useful for developing a reliable clinical application [2].

The study pooled the dataset of microarrays from different research teams in the Gene Expression Omnibus (GEO) database in order to increase sample size, sample heterogeneity, and statistical power in the hope of addressing the issue of insufficient sample size presented in previous studies. Four methods, Prediction Analysis of Microarray (PAM), Artificial Neural Network (ANN), Classification and Regression Trees (CART), and C5.0, were employed to analyze the variations in gene expression between colorectal tumors and normal mucosa tissues in order to screen for significant genes.

## 2. Methods

### 2.1. Microarray Data Sources

The microarray gene expression data are from searches using “colon cancer” and “human [organism]” and “expression profiling by array [dataset type]” as the keywords in the GEO database of the National Center for Biotechnology Information (NCBI). The eligible criteria were as follows: (1) the examined samples were frozen tissue sections of normal human colorectal mucosa, primary colorectal cancer, or hepatic metastases from colorectal cancer; (2) the microarray platform used was limited to single-color, whole genome gene chips from Affymetrix; and (3) the data were presented as gene expression level. The exclusion criteria were as follows: (1) data from cultured cell lines or other in vitro assays; (2) datasets without the original gene expression level data files; and (3) those with redundant subdatasets. A total of 190 GEO series (GSE) datasets were finally 174 excluded, leaving 16 independent datasets for analysis, which are as follows: GSE4045, GSE4107, GSE4183, GSE5851, GSE8671, GSE9348, GSE1096, GSE12630, GSE12945, GSE13067, GSE13294, GSE13471, GSE15960, GSE17538, GSE18105, and GSE14333. The total number of examined tissues was 1,274, including 88 normal mucosal tissues and 1,186 colorectal tumor tissues (53 adenoma tissues, 521 adenocarcinoma tissues, 533 primary colorectal tumor tissues, and 79 hepatic tissues with metastatic colorectal tumors). The data set is unbalanced among groups of adenoma, adenocarcinoma, carcinoma, metastasis, and normal tissues having 53, 521, 533, 79, and 88 samples, whereas, in training set, candidate genes modeling two remaining groups between cases and controls must have accuracies more than 88/(88 + 53) = 0.6241, 521/(88 + 521)= 0.8555, 533/(88 + 533) = 0.8583, and 88/(88 + 79) = 0.5269, respectively.

**Figure 1:**
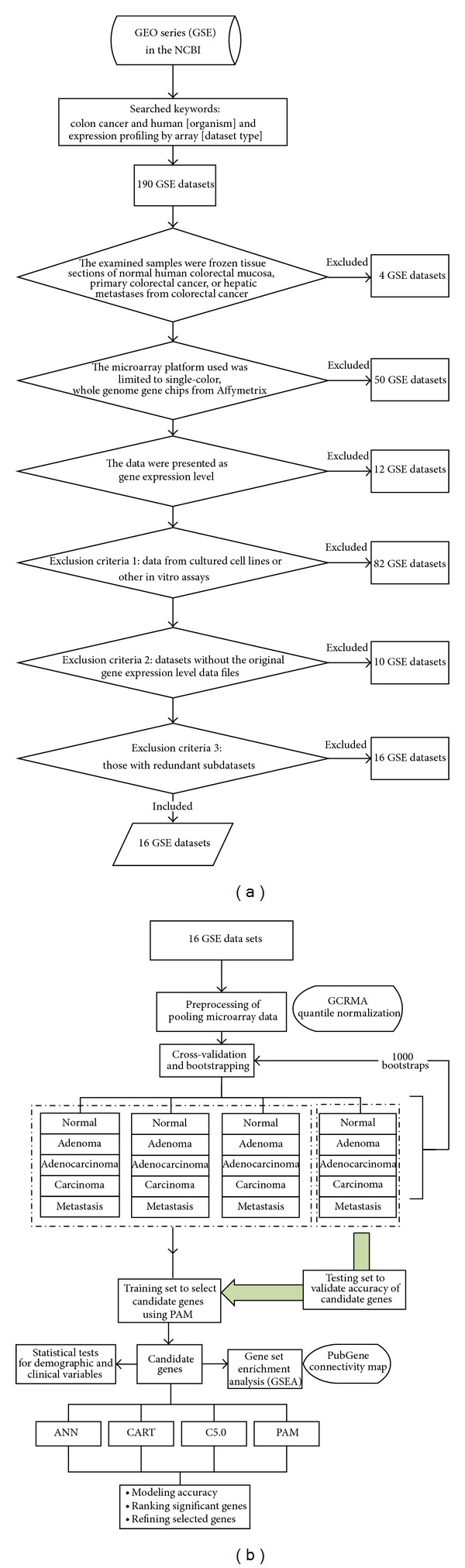
(a) The research process of flow chart diagram for the datasets with analysis. (b) Diagram of the methods used to identify candidate genes and establish prediction models.

All microarray data of examined tissues were produced from primary surgical cases without chemotherapy. For adjusting metastatic spread, all the cases were divided into five groups based on pathological tissue types: normal mucosal tissues (nm), adenoma (ad), adenocarcinoma (ac), primary tumor (unknown type, primary carcinoma; cn), and liver metastases (mt). More clinical factors of stage, grade, microsatellite instability (MSI), microsatellite stability (MSS), sites, race, gender, and age were adjusted for gene ordering and secondary genetic screening in the multinomial and multivariate logistic regression. The research process of flow chart diagram for the pooled datasets with analysis included in the study is shown in Figures 1(a) and 1(b).

### 2.2. Preprocessing of Pooling Microarray Data

In Figure 1(b), we used the GC Robust Multiarray Average (GCRMA) method and R language software [8] to remove the chip background associated with the microarray gene expression levels. The expression levels of the probe sets were converted into gene expression levels. Because the probe expression levels showed a skewed distribution, the median probe expression level was selected to represent the gene expression level. Therefore, Bolstad et al. [9] proposed the GCRMA to adjust the affinity among nucleotides because of different binding strengths between GC and AT rather than the RMA provided by the Affymetrix Console, as the latter is designed for processing Affymetrix chips. The preprocessed microarrays were first performed within-study normalization using GCRMA and then calculated gene level estimates before combining the different studies and only keeping genes available on all arrays. Afterwards, the preprocess also did between-study normalization.

For instance, the HG-U133A gene chip used in this study is comprised of 22,283 probes that cover 14,713 genes. Each gene is covered by 1–14 probes. Of the 14,713 genes, 5,107 (38%) are covered by more than two probe combinations. For genes covered by multiple probe combinations, this study adopted the median method. For example, when the expression level of the HFE gene was reflected by the levels of 13 probes, the level of the seventh (the median number) probe was chosen to represent the expression of the HFE gene.

Affymetrix chips were HG-U133A, HG-U133A-2, and HG-U133-Plus-2. Among these 3 types of chips, the corresponding numbers of genes were 14,713, 14,704, and 33,727, after the conversion that each single gene can map several probes, respectively. In the present study, the quartile normalization of all gene expression values was performed before combining 16 GSEs. The probes were merged to obtain the expression levels of 14,698 genes. The normalization can eliminate the systematic variations among studies [10].

### 2.3. Primary Screening of Candidate Genes

The candidate genes of colorectal tumors and normal mucosal tissues were selected from the primary screening of the 14,698 genes using PAM, followed by the construction of a classification model, determination of gene order of importance, and secondary gene screening using ANN, CART, C5.0, and PAM. decision trees were constructed using CART and C5.0 for intragroup comparison of gene order of importance.

The ad, ac, cn, and mt groups were subjected to pairwise comparison of gene expression with the nm group to identify genes that were differentially expressed at different cancer stages. The bootstrapping rounds were used to avoid the poor extrapolation of the selected candidate genes that the proposed analysis is unbiased and would not lead to over-fitting. Three-fourths of the samples in each pair of comparison groups were randomly selected as the model-training group for PAM, and the remaining fourth served as the model-testing group for an independent set of samples that is left for final validation. Also, it will be interesting to see the results of the current method under the null hypothesis of nor predictive signal, which can be achieved by label permutation. The threshold criteria for gene selection by PAM were that the candidate genes were with the minimum classification errors, which number was lower than 100.

### 2.4. Construction of the Four Model Analysis

Using the significant genes from the primary screening, four classification models were constructed for colorectal tumor tissues (ad, ac, cn, and mt) and normal mucosal tissues (nm) for the secondary screening of significant genes and determination of the gene order of importance. Completely separating training and validation data sets is a crucial step in classifier development (Grade et al., 2007; Simon, Radmacher, Dobbin, and McShane, 2003). It is very important that none of the samples in the validation dataset has been used in any part of the training, for example, for feature selection. In Figure 1(b), bootstrapping was performed 1,000 rounds for the training of each model with three-fourths of the samples, and the remaining fourth for the model-testing group for an independent set of samples that is left for final validation. Also, it will be interesting to see the results of the current method under the null hypothesis of nor predictive signal, which can be achieved by label permutation. The related settings of the four analysis models are shown in Supplementary Table  1 (see Supplementary Materials available online at http://dx.doi.org/10.1155/2014/634123). The introduction of the four analysis methods is as follows. The Clementine 10.1 was performed for ANN, C5.0, and CART and the R was used for PAM.

### 2.5. PAM

PAM uses nearest shrunken centroids as its algorithm. It can eliminate noise signal [11], control the false discovery rate (FDR), and select the best candidate gene set. PAM method has been reported to perform better with fewer genes than the original methods in previous studies [11, 12]. PAM was employed for the screening for primary candidate genes, secondary gene selection, and model construction. Tibshirani et al. [13] reanalyzed the microarray data of leukemia from Golub et al. [14] and concluded 43 from 96 genes via PAM compared to that Khan et al. [15] who concluded them via ANN. Meanwhile, the FDR was reduced from 4 to 2 over 34 classifications.

### 2.6. ANN

ANN needs to go through repeated training and learning to construct good classification models. It has the following advantages: (1) it is suitable for the analysis of highly dimensional and uncommon data; (2) it can accept missing values and process noise of the information. Its limitations are as follows: (1) it is necessary to avoid over-training the model and (2) it has a nontransparent solution process known as a “black box” (3). ANN were trained by the setting items of method with quick back propagation, prevent overtraining with 80% samples, set random seed-seed with 720925, stop on with default, optimize with speed. The Quick back propagation with producing smaller hidden layer and random seed 720925 with randomization for avoiding over-fitting were default in ANN on Clementine 10.1.

### 2.7. C5.0 and CART

Decision trees are models with tree-like structures. Trimming the branches can solve the problem of over-training. In general, larger decision trees are more expressive and may have more predictive power, but the smaller a decision tree is, the stronger its simplicity is [16]. The construction of CART was based on a Gini index, with the best independent variable chosen for the binary cut in each branch. Therefore, each independent variable (field) is likely to be used repeatedly at different nodes. C5.0 was developed gradually from C4.5 and ID3 and is similar to the CART method. The major difference is that the construction of its decision tree is based on information gain [17].

### 2.8. Ordering Method for Gene Importance

The expression values of the 55 genes were analyzed in the models constructed with ANN, CART, C5.0, and PAM, and the contribution level of each gene to the classification of colorectal tumors and normal mucosal tissues was obtained, which was designated as gene importance.

CART, C5.0, and PAM used the number of times each gene was selected as a predictor in 1,000 bootstrapping rounds. A higher number of times indicated a higher importance for the gene. ANN listed the relative importance values of the input gene variables in the classification model, with larger values representing higher contribution levels. In addition to the ordering methods above, PAM and C5.0 also used the centroids values, as well as the location of a gene, as a node, in the decision tree, respectively, to calculate gene importance. The higher absolute values of centroids and a node, which are closer to the root of a decision tree (i.e., the first split point), represent the higher importance for the gene. All the index levels of gene importance in each model were ranked and normalized into percentile ranked score (RS%). For precise settings, see the Supplementary Table  2.

### 2.9. Functional Pathway Analysis

The use of pooled GEO studies was secondary because only microarray data was available. Testing 55 genes in any experimental model would be beneficial for colon cancer biology. Therefore, the present study analyzed the functional pathways that are related to the tumor genesis of colorectal cancer using GSEA software version 2.07. The GSEA MSigDB provides a collection of annotated gene sets based on different sources of information, for example, gene ontology, pathways, or motifs [10]. Input variables were the expression values of the 14,698 genes in colorectal tumor tissues and normal mucosal tissues. The related settings and gene ordering results are shown in the Supplementary Tables  2 and 3. (*ANN*: the ANN model listed the relative importance values (RI) of the input gene variables in the classification of colorectal tumors and normal mucosal tissues, with larger values representing higher contribution levels. The relative importance values were ranked with 1 point for the lowest value, 2 points for the next lowest value, and so on. Identical ranked scores (RS) were given to the genes with the same RI value. Finally, each gene's RS was divided by the highest RS in order to obtain the percentile ranked score (RS%).* CART*: the gene importance ordering method in the CART model was used to calculate the number of times each gene was selected as a node in the decision tree in the analysis with 1,000 repeated samplings. A higher number of times selected indicated a higher importance for the gene. The genes were ranked based on the number of times they were selected as a significant gene, with 1 point for the lowest number of times, 2 points for the next lowest number of times, and so on. The same ranked score (RS) was given to the genes with the same Sig value. Finally, the RS of each gene was divided by the highest RS to obtain the RS%.* PAM*: one of the PAM gene importance calculation methods is similar to CART. However, in addition to using Sig to calculate importance, PAM used the centroids values (Cen) to calculate gene importance. The detailed calculation method using Cen was as follows. The absolute values were obtained for the averages of the centroids from the PAM analysis results after taking 1,000 repeated samples for the four group pairs (ad/nm, ac/nm, cn/nm, and mt/nm). Next, the sum of the absolute values for each gene was ranked, with 1 point for the lowest value, 2 points for the next lowest value, and so on. The same RS was given to identical centroids values. Finally, the RS of each gene was divided by the highest RS to obtain the RS%.* C5.0*: the calculation method is based on the number of times each gene is selected, similar to CART, as well as node location. The latter method was that when a gene, as a node, is closer to the root of a decision tree (i.e., the first split point), it has a higher RS, while it has a lower RS, when it is closer to the tip of the branches, and so on. The RS% calculation method is the same as that of CART. The ranked scores were converted to percentiles in order to make it possible to compare the order of gene importance obtained from these four analysis methods.)

## 3. Results

The age distribution indicated a higher proportion of colorectal cancer in people over 60 years old. No difference was found in the location of collected tissues or genders between the two groups (Table 1).

### 3.1. Primary Genetic Screening

The PAM screens the candidate genes with highly statistical significances by bootstrapping and cross-validation and then classification algorithms implement models with selected genes that can discriminate between two groups among cancers and controls. The results demonstrate how consistent different methods of data mining perform and select candidate genes among ANN, decision trees, and PAM according to correlation coefficients via ranking statistical significances of 55 genes.

**Figure 2:**
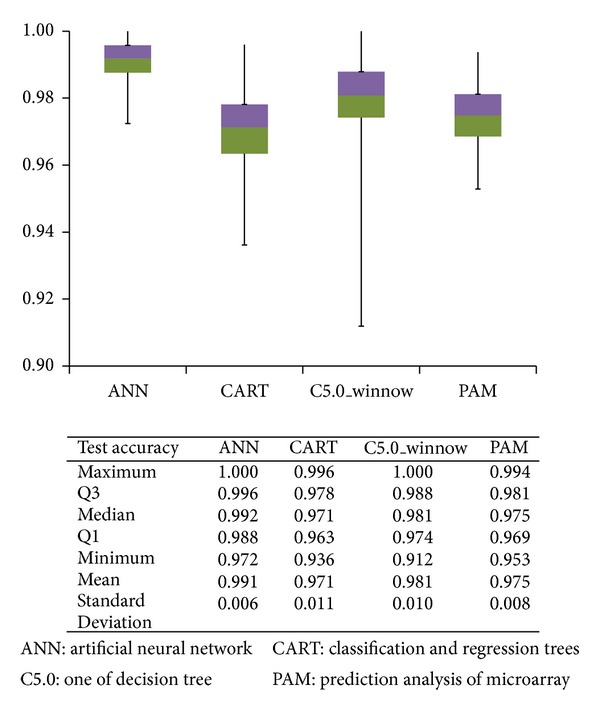
Test accuracy rates of 4 approaches in 1,000 bootstrapping rounds for classifying colorectal tumors and normal mucosal tissues.

Significant genes were found in the ad/nm, ac/nm, cn/nm, and mt/nm combination groups, respectively, and a total of 55 significant genes were identified (Table 2). The repetitions in the four comparison groups and the variations of accuracy were very small and the average accuracy was above 0.95 (Figure 2). Less than 18 genes were screened each time, and the FDR value of each analysis was close to 0, which indicates that the probability of false positive results for gene significance was close to 0 (see the Supplementary Table  4 and Supplementary Figure  1).

These 55 genes are mainly localized and functional at the cell surface and at tight junction. Their molecular functions are related to transporter activity, binding, catalytic activity, enzyme regulator activity, and gelatinase activity. The biological processes that these genes are involved in mainly include biological adhesion, signaling, transporter, metabolic process, insulin secretion, and biological regulation (see the Supplementary Tables  5–7).

### 3.2. The Construction of Classification Models

The mean values for the test accuracy reached 0.97 or above in the classification of normal and colorectal cancer cases. ANN and C5.0 exhibited the best performances, and ANN showed the best model stability (Figure 2). CART required the least number of gene variables, two genes (median value), for each analysis. Less than 8 genes were selected in all the models on average, which suggests that less than 8 genes can effectively classify normal and colorectal cancer cases (see the Supplementary Table  8).

The results in Table 3 demonstrate how consistent different methods of data mining perform and select candidate genes among ANN, decision trees, and PAM according to correlation coefficients via ranking statistical significances of 55 genes. The order of the importance of the 55 genes reported by C5.0Δ and C5.0_importance showed very good consistency with a Spearman's correlation coefficient of 0.73 (*P* < 0.001) (Table 3). PAMΔ and PAM_centroid gave the exact same ranking order for the importance of the 55 genes. The results suggested that, in the repeated sampling analysis, results of classification of colorectal tumors and normal tissues via C5.0 and PAM, the number of times of a gene is deemed significant, can be generally used as a reference for the ranking order for gene importance; the higher the number of times a gene is called significant, the more important the gene is as a classification dependent variable.

CARTΔ and C5.0Δ and C5.0_importance, an internal control group, had the similar importance orders of 55 genes (Spearman's correlation coefficient between 0.62 and 0.75 and *P* < 0.001). ANN showed a significant correlation with CARTΔ and C5.0Δ but had relatively low correlation coefficients in the range of 0.42–0.48 (Table 3). In summary, a poor consistency in gene importance rank ordering was observed among the different methods.

### 3.3. Gene Ordering and Secondary Genetic Screening

**Figure 3:**
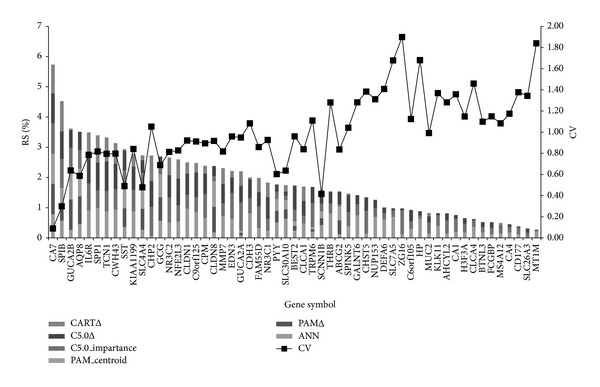
Stacked bar chart of the gene importance of 55 genes. The percentile ranks of scores (RS%) of 55 genes were derived from PAM, ANN, CART, and C5.0 methods. The higher RS% represents that the gene was more important in the classification of colorectal tumors and normal mucosa tissues. CV: coefficient of variance. The RS% of genes in different methods was calculated by four approaches noted with the suffix, Δ, importance, centroid, and #. Symbols represent the way to calculate RS%. Δ: the significant times of each gene in 1,000 times of bootstrapping. Importance: the node location of genes in decision trees. Centroids: centroid values of each gene in PAM. #: the relative importance values in ANN.

In Figure 1(b), eight genes were selected by best ranking for modeling *P* values from bootstrapping ANN, decision tree, and PAM. The results were shown in Figures 2 and 3. Models sum of RS% was calculated, which was generally inversely proportional to the coefficient of variance (CV) value. The more important the gene is, the more consistent the ranking orders are among the various models. The top eight genes after ranking by RS% sum were selected, and they are* CA7*,* SPIB*,* GUCA2B*,* AQP8*,* IL6R*,* SPP1*,* TCN1*, and* CWH43* (Figure 3). The RS% obtained by all of the methods was 0.5 or greater for* CA7*,* SPIB*, and* CWH43* (see the Supplementary Table  3). All genes except* TCN1* remained significant after individual controlling for gender, race, tissue location or age, and* CA7*,* SPIB*, and* CWH43* were still significant when the four confounders were controlled for simultaneously. Except for* SPP1*, which did not show a correlation with microsatellite stability, the other genes all exhibited significant correlation after logistic regression of individual variables, with OR values ranging from 0.49 to 1.12. Of the eight genes, only* SPP1* was significantly associated with cancer stage. The higher the expression level of* SPP*, the more advanced the cancer stage was (see the Supplementary Tables  9 and 10). The multivariate logistic regression was performed for acquiring odds ratios of 8 genes expression of each of the eight genes adjusted by demographics in the four different groups. These odds ratios allow comparisons of patterns of expression changes for each of the genes; for example, whether gene expression increases (odds ratio > 1) for groups with more severe lesions or whether there is only a difference between normal and disease tissue.

The Supplementary Table  10 showed that* DEFA6*,* TCN1*, and* KLK11* are upregulated and* NR3C1* and* THRB* are downregulated in adenoma, which can be earlier screening markers. The genes of* ABCG2*,* CA7*,* HP*,* KIAA1199*, and* CLDN1* are upregulated and* CA4*,* CHP2*,* CHST5*,* CLCA1*,* CLCA4*,* CLDN8*,* FAM55D*,* H3F3A*,* MUC2*, and* NR3C2* are downregulated in carcinoma and metastases, which can be prognosis monitoring markers. The markers are regulated consistently with same direction comparing the present study, Cardoso et al. 2007 and Chan et al. 2008 [4, 7], the gene family present genes related with the selective markers that are not mapped the same in literature.

Of these eight genes,* AQP8*,* SPIB*,* SPP1*, and* TCN1* are GO biological process annotated as transporters;* CA7* and* GUCA2B* are annotated as being involved in biological regulation and signaling, respectively;* IL6R* is annotated as being part of the immune system process, while no annotation was available for* CWH43*. As for GO molecular function annotation,* CA7*,* IL6R*, and* SPP1* are annotated as having gelatinase activity;* AQP8*,* SPIB*, and* TCN1* are annotated as being capable of binding;* GUCA2B* is annotated as a regulator of enzyme activity, and* CWH43* is not annotated.

### 3.4. The GSEA Analysis of Functional Pathways

The GO functional results were similar to the results from the GSEA analysis with the 55 genes, suggesting these 55 genes are indeed located in the significant functional pathways that affect the tumor genesis of colorectal cancer.

The primary GO molecular function annotation is binding (nucleic acid binding, chromatin binding, and peptide binding). Although peptide binding was downregulated in colorectal tumor tissues, the other types of binding were all upregulated. The activities of genes involved in channel regulation signal transduction and transportation were suppressed in colorectal cancer. Of the GO biological processes, cell cycle, DNA replication, DNA metabolic process, and the glutamate signaling pathway were upregulated in colorectal cancer, while response to stimulus (feeding behavior) and lipid metabolism was downregulated (see the Supplementary Table  11).

## 4. Discussion

The present study aimed at finding possible marker gene sets for colorectal cancer by using a two-step complex bioinformatical analysis. The in silico gene expression analysis results in a low numbered gene set that could be a potent classificatory set for CRC. In a biological point of view, the methods and the results are valid; however, the verification of the given gene set on an independent sample set would be necessary, even at protein level. Biostatistically, it would be interesting to know whether the selected, highly discriminative genes are also present in the excluded GSE datasets.

### 4.1. Comparison of Analysis Methods

Four models performed very well at classifying colorectal tumors and normal mucosal tissues with the average test accuracy rates above 0.97. ANN showed the best classification performance, with an average test accuracy of 0.99 ± 0.01. CART required the fewest genes, requiring an average of 1.7 genes to reach an average test accuracy of 0.97. C5.0 had the best accuracy with the fewest genes among the four methods.

In general, except for C5.0 and CART, a poor consistency in gene importance rank ordering was observed among the different methods. Lee et al. [11] observed the same results and found that, in general, more sophisticated classifiers give better performances than do classical methods. However, the present study found that genes of higher importance showed lower variation in rank ordering.

### 4.2. Gene Overlapping

Among 55 selected genes, 13 overlapped with significant genes mentioned in two review articles on gene expression analysis in colorectal cancer [4, 7]. The overlap rate, 13/55 (24%), was not particularly high. However, if the seven genes in the same gene family that overlapped are included, the overlap rate increases to 36%. Compared to the 40 genes identified by Verkman et al. [18], there are 21 overlapping genes, and the overlap rate is 21/55 (38%). These overlapping genes are* ABCG2*,* AQP8*,* CA1*,* CA4*,* CA7*,* CD177*,* CDH3*,* CLCA4*,* CLDN8*,* EDN3*,* FCGBP*,* GCG*,* GUCA2A*,* GUCA2B*,* KIAA1199*,* MMP7*,* MS4A12*,* MT1M*,* MUC2*,* PYY*,* SLC26A3*,* SLC4A4*,* SPIB*,* SST*, and* ZG16*.

### 4.3. Top Eight Genes

Gelatinase activity was one major GO annotation of these eight genes (*CA7*,* SPIB*,* GUCA2B*,* AQP8*,* IL6R*,* SPP1*,* TCN1*, and* CWH4*) and is related to tumor progress and metastasis. Its expression was elevated in advanced or metastatic tumor tissues [19–22]. The following is an introduction of the eight genes important for the classification of colorectal cancer and normal tissues.

#### 4.3.1. *CA7* (Carbonic Anhydrase VII)

*CA7* was indicated as an important suppressor gene for the classification of normal and colorectal cancer tissues in this study. Among the members of the CA isozyme family, CA2 [7], CA9, and CA12 have been shown to be related to tumor genesis [12]. However, no reports have been published on the relationship between* CA7* and colorectal cancer.

#### 4.3.2. *SPIB* (Spi-B Transcription Factor)

The Spi-B transcription factor protein is a member of the ETS transcription factor family, which inhibits neoangiogenesis, tumor progression, and metastasis [23, 24]. Currently, no relevant studies have demonstrated a potential mechanism underlying the relationship between* SPIB* and the tumor genesis of colorectal cancer.

#### 4.3.3. *GUCA2B* (Guanylate Cyclase Activator 2B, Uroguanylin)

Uroguanylin (GUCA2B) and guanylin (GUCA2A) are very close in structure and biological functions. They are responsible for signal transduction that regulates the transport and secretion of liquids and electrolytes in the gastrointestinal tract during digestion [25, 26]. The suppression of this function was found to promote tumor genesis [27, 28].

#### 4.3.4. *AQP8* (Aquaporin 8)

Aquaporin are responsible for water absorption and excretion in the gastrointestinal tract [29]. They play important roles in the metastasis and proliferation of cancer cells [18]. Fischer et al. discovered that* AQP8* expression was only found in the columnar surface of epithelial cells; little or no expression of* AQP8* was observed in the colonic crypts or tumor cells [19], which is also consistent with Galamb et al. [20].

#### 4.3.5. *IL6R* (Interleukin 6 Receptor)

*IL6R* is primarily involved in the immune response, inflammation, and hematopoiesis [21] and was downregulated in the present study. The low expression level of sIL-6R in colorectal cancer tissues was correlated with disease progression, and it can serve as an independent factor for prognosis prediction [22].

#### 4.3.6. *SPP1* (Secreted Phosphoprotein 1, Osteopontin)

Osteopontin protein can be used as a marker of tumor progression for breast cancer [30], lung cancer [31], and prostate cancer [32]. The expression level of osteopontin is remarkably increased in the blood samples from advanced colorectal cancer [33] and has been considered to be a marker for colorectal cancer progression [34].

#### 4.3.7. *TCN1* (Transcobalamin 1)

*TCN1* is a regulator of the Wnt/beta-catenin pathway and enhances the expression of the target genes of beta-catenin, leading to cancer progression and outcome deterioration. In gastric cancer studies,* TCN1* was significantly correlated with cancer stage, poor cell differentiation, lymph node metastasis, and a poor prognosis [35].

#### 4.3.8. *CWH43* (Cell Wall Biogenesis 43 C-Terminal Homolog)

*CWH43* has been reported to contribute to the cell wall integrity of Saccharomyces cerevisiae [36]. However, at present, no studies have reported its relationship with human cancer. The expression of* CWH43* was downregulated in colorectal tumor tissues (fold change = −4.59).

## 5. Conclusion

This study adopted a two-tier genetic screen, which not only reduced the number of candidate significant genes but also resulted in an impressively nearly 100% test accuracy. This analysis method can be applied to future pooled microarray studies. The more important genes exhibit a more consistent ranking order among the different methods used.* CA7*,* TCN1*, and* CWH43* are novel genes, but have not been previously reported as related to colorectal cancer. Further researches will help us better understand their roles in colorectal cancer.

## Supplementary Material

Supplementary materials contain the details about settings of models, parameters of selected genes, of optimizing selected number of genes and gene ontological pathways.

## Figures and Tables

**Table 1:** Descriptive statistics of study samples.

Variables	Normal mucosa		Colorectal tumors		Logistic regression	
	*n*	%	*n*	%	OR	*P* value
Gender (*n* = 759)						
Female	10	48	331	45	1	
Male	11	52	407	55	1.12	0.8
Age (*n* = 722)						
≤60	17	81	240	33	1	
>60	4	19	461	62	8.16	∗∗∗
Race (*n* = 1,274)						
European	14	16	321	27	1	
Han	38	43	177	15	0.21	∗∗∗
Australia	32	36	389	33	0.54	0.05
USA	4	5	299	25	3.26	∗
Location of tissues (*n* = 272)						
Proximal	5	11	32	14	1	
Distal	42	89	193	86	0.72	0.52

OR: odds ratio; _ _*<0.05; _ _***<0.001. Proximal position: cecum, ascending colon, hepatic flexure, transverse colon, and splenic flexure. Distal position: descending colon, sigmoid colon, and rectum.

**Table 2:** 55 differential expressed genes in colorectal tumors of the primary screening.

Accession number	Gene symbol	Gene name	Chromosome	Fold change*
HGNC: 74	*ABCG2 *	ATP-binding cassette, subfamily G (white), member 2	4q22	−6.26
HGNC: 22204	*AHCYL2 *	Adenosylhomocysteinase-like 2	7q32.1	−5.82
HGNC: 642	*AQP8 *	Aquaporin 8	16p12	−6.56
HGNC: 17107	*BEST2 *	Bestrophin 2	19p13.2	−4.52
HGNC: 1143	*BTNL3 *	Butyrophilin-like 3	5q35.3	−6.03
HGNC: 21214	*C6orf105 *	Chromosome 6 open reading frame 105	6p24.1	−7.31
HGNC: 28180	*C9orf125 *	Chromosome 6 open reading frame 105	9q31.1	−4.23
HGNC: 1368	*CA1 *	Carbonic anhydrase I	8q21.2	−6.22
HGNC: 1375	*CA4 *	Carbonic anhydrase IV	17q23	−3.95
HGNC: 1381	*CA7 *	Carbonic anhydrase VII	16q22.1	−5.39
HGNC: 30072	*CD177 *	CD177 molecule	19q13.2	−4.65
HGNC: 1762	*CDH3 *	Cadherin 3, type 1, *P*-cadherin (placental)	16q22.1	5.49
ENSG00000166869	*CHP2 *	Calcineurin B homologous protein 2	16p12.2	−6.23
HGNC: 1973	*CHST5 *	Carbohydrate (*N*-acetylglucosamine 6-O) sulfotransferase 5	16q22.3	−3.83
HGNC: 2015	*CLCA1 *	Chloride channel accessory 1	1p22.3	−5.68
HGNC: 2018	*CLCA4 *	Chloride channel accessory 4	1p31-p22	−4.08
HGNC: 2032	*CLDN1 *	Claudin 1	3q28-q29	5.36
HGNC: 2050	*CLDN8 *	Claudin 8	21q22.11	−3.69
HGNC: 2311	*CPM *	Carboxypeptidase M	12q14.3	−3.64
HGNC: 26133	*CWH43 *	Cell wall biogenesis 43 C-terminal homolog (*S. cerevisiae*)	4p11	−4.59
HGNC: 2765	*DEFA6 *	Defensin, alpha 6, Paneth cell-specific	8p23.1	5.95
HGNC: 3178	*EDN3 *	Endothelin 3	20q13.2-q13.3	−4.27
HGNC: 23117	*FAM55D *	Family with sequence similarity 55, member D	11q23.2	−6.18
HGNC: 13572	*FCGBP *	Fc fragment of IgG binding protein	19q13.1	−5.52
HGNC: 4128	*GALNT6 *	UDP-*N*-acetyl-alpha-*D*-galactosamine:polypeptide *N*-acetylgalactosaminyltransferase 6 (GalNAc-T6)	12q13	3.56
HGNC: 4191	*GCG *	Glucagon	2q36-q37	−6.1
HGNC: 4682	*GUCA2A *	Guanylate cyclase activator 2A (guanylin)	1p35-p34	−4.32
HGNC: 4683	*GUCA2B *	Guanylate cyclase activator 2B (uroguanylin)	1p34-p33	−6.62
HGNC: 4764	*H3F3A *	H3 histone, family 3A	1q41	−3.86
HGNC: 5141	*HP *	Haptoglobin	16q22.1	11.72
HGNC: 6019	*IL6R *	Interleukin 6 receptor	1q21	−3.46
HGNC: 29213	*KIAA1199 *	KIAA1199	15q24	4.43
HGNC: 6359	*KLK11 *	Kallikrein-related peptidase 11	19q13.33	4.65
HGNC: 7174	*MMP7 *	Matrix metallopeptidase 7 (matrilysin, uterine)	11q21-q22	6.75
HGNC: 13370	*MS4A12 *	Membrane-spanning 4-domains, subfamily A, member 12	11q12	−10.66
HGNC: 14296	*MT1M *	Metallothionein 1M	16q13	−3.94
HGNC: 7512	*MUC2 *	Mucin 2, oligomeric mucus/gel-forming	11p15.5	−7.23
HGNC: 7783	*NFE2L3 *	Nuclear factor (erythroid-derived 2)-like 3	7p15.2	3.02
HGNC: 7978	*NR3C1 *	Nuclear receptor subfamily 3, group C, member 1 (glucocorticoid receptor)	5q31.3	−3.2
HGNC: 7979	*NR3C2 *	Nuclear receptor subfamily 3, group C, member 2	4q31.1	−5.08
HGNC: 8062	*NUP153 *	Nucleoporin 153kDa	6p22.3	5.9
HGNC: 9748	*PYY *	Peptide YY	17q21.1	−4.35
HGNC: 10600	*SCNN1B *	Sodium channel, nonvoltage-gated 1, beta	16p12.2-p12.1	−3.28
HGNC: 3018	*SLC26A3 *	Solute carrier family 26, member 3	7q31	−5.59
HGNC: 25355	*SLC30A10 *	Solute carrier family 30, member 10	1q41	−5.69
HGNC: 11030	*SLC4A4 *	Solute carrier family 4, sodium bicarbonate cotransporter, member 4	4q21	−5.14
HGNC: 11063	*SLC7A5 *	Solute carrier family 7 (cationic amino acid transporter, y+ system), member 5	16q24.3	4.05
HGNC: 11242	*SPIB *	Spi-B transcription factor (Spi-1/PU.1 related)	19q13.3-q13.4	−4.24
HGNC: 15464	*SPINK5 *	Serine peptidase inhibitor, Kazal type 5	5q32	−4.31
HGNC: 11255	*SPP1 *	Secreted phosphoprotein 1	4q22.1	9.69
HGNC: 11329	*SST *	Somatostatin	3q28	−5.84
HGNC: 11652	*TCN1 *	Transcobalamin I (vitamin B12 binding protein, R binder family)	11q11-q12	8.66
HGNC: 11799	*THRB *	Thyroid hormone receptor, beta (erythroblastic leukemia viral (v-erb-a) oncogene homolog 2, avian)	3q24.2	−4.25
HGNC: 17995	*TRPM6 *	Transient receptor potential cation channel, subfamily M, member 6	9q21.13	−4.85
HGNC: 30961	*ZG16 *	Zymogen granule protein 16 homolog (rat)	16q11.2	−3.94

*equation: fold change = log_2_⁡(*g*_crc_/*g*_nm_); *g*_crc_: the average gene expression in colorectal tumors; *g*_nm_: the average gene expression in normal mucosal tissues.

**Table 3:** Spearman's correlations of ranking orders of 55 significant genes among the methods of PAM, ANN, CART, and C5.0.

	Spearman's correlation	ANN	CARTΔ	C5.0Δ	C5.0_importance	PAM_centroid	PAMΔ
ANN	*r*_*s*_	1					
CARTΔ	*r*_*s*_	0.42**	1				
C5.0Δ	*r*_*s*_	0.48***	0.75***	1			
C5.0_importance	*r*_*s*_	0.24	0.62***	0.73***	1		
PAM_centroid	*r*_*s*_	0.09	0.01	0.18	0.1	1	
PAMΔ	*r*_*s*_	0.09	0.01	0.18	0.1	1.00***	1

*P value < 0.05,  **P value < 0.01,  ***P value < 0.001.

## Abbreviations

ACC: Accuracy
AUC: Area under the curve
DT: Decision tree
LR: Logistic regression
ANN: Artificial Neural Network
GEO: Gene Expression Omnibus
