# Supplementary material for: Gene Expression Profiling of Colorectal Tumors and Normal Mucosa by Microarrays Meta-Analysis Using Prediction Analysis of Microarray, Artificial Neural Network, Classification, and Regression Trees
Source: Dis Markers. 2014 May 19;2014:634123. doi: 10.1155/2014/634123 (PMC4055246; doi:10.1155/2014/634123)
Supplement: Supplementary file 1 — Supplementary materials contain the details about settings of models, parameters of selected genes, of optimizing selected number of genes and gene ontological pathways. [file 634123.f1.pdf]

Supplementary materials.

Table 1 The Settings of ANN,CART, C5.0 and PAM methods

	ANN	C5.0	CART	PAM
**Setting items**	Method: Quick backpropagation	Use partitioned data: True	Use partitioned data: True	Threshold: The candidate genes were with the minimum classification errors, which number was lower than 100.
	Prevent overtraining: 80% Samples	Output type:<br>Decision tree<br>Use boosting	Build: Model	
	Set random seed-Seed:720925	Mode: Expert	Maximum tree depth-Levels below root:7	
	Stop on: Default	Pruning severity:0		
	Optimize: Speed	Minimum records per child branch:2		
		Use global pruning: True		
		Winnow attributes: True		

Table 2 The settings of the Gene Set Enrichment Analysis method

Items	Settings
**Number of permutations**	1000
**Collapse dataset to gene symbols**	False
**Permutation type**	phenotype
**Enrichment statistic**	weighted
**Metric of ranking genes**	Signal2Noise
**Max size**	500
**Min size**	15

Footnote 1：The gene sets of reference for Gene Set Enrichment Analysis (GSEA) are derived from MSigDB (figure shown in the below). In the present study, C1 positional gene sets（C1_ALL）, C2:canonical pathways（C2_CP）, genemapp（C2_genmapp）, kegg（C2_kegg）, C5:GO biological process（C5_BP）, GO cellular component(C5_CC), and GO molecular function（C5_MF） were selected. The definitions of significant gene sets analyzed by GSEA method were 1）NOM p-val<0.05 and 2）Familywise-error rate (FWER)<0.25.NOM p-val is the statistical significance of the enrichment score. The nominal p value is not adjusted for gene set size or multiple hypothesis testing; therefore, it is of limited use in comparing gene sets. FWER is a more conservatively estimated probability that the normalized enrichment score represents a false positive finding.

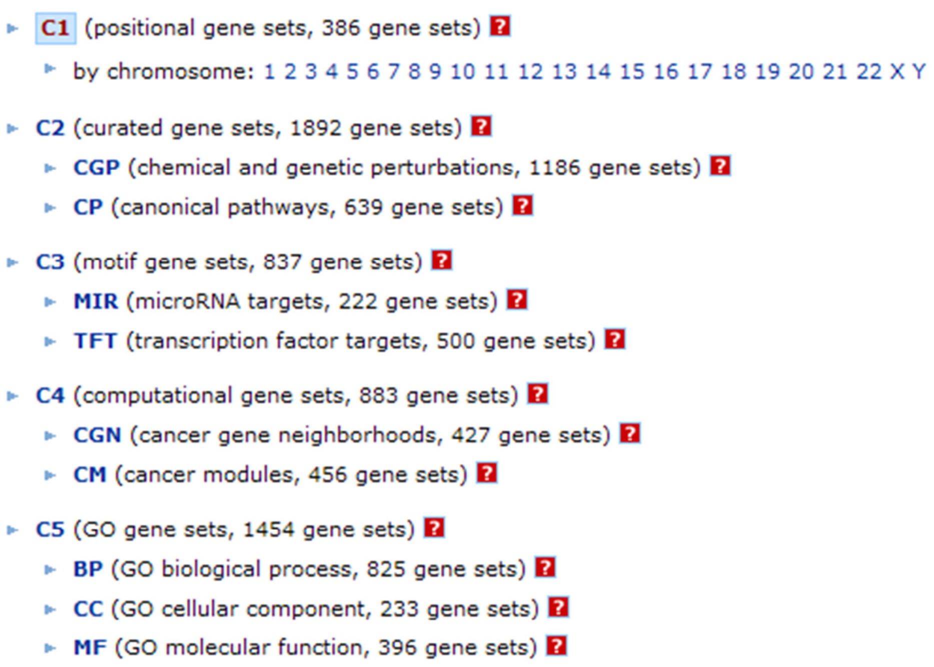

Footnote 2：The detailed ordering methods of gene importance were as follows.

ANN

The ANN model listed the relative importance values (RI) of the input gene variables in the classification of colorectal tumors and normal mucosal tissues, with larger values representing higher contribution levels. The relative importance values were ranked with 1 point for the lowest value, 2 points for the next lowest value and so on. Identical ranked scores (RS) was given to the genes with the same RI value. Finally, each gene's RS was divided by the highest RS in order to obtain the percentile ranked score (RS%)

CART

The gene importance ordering method in the CART model was used to calculate the number of times each gene was selected as a node in the decision tree in the analysis with 1,000 repeated samplings. A higher number of times selected indicated a higher importance for the gene. The genes were ranked based on the number of times they were selected as a significant gene, with 1 point for the lowest number of times, 2 points for the next lowest number of times and so on. The same ranked score (RS) was given to the genes with the same Sig value. Finally, the RS of each gene was divided by the highest RS to obtain the RS%.

PAM

One of the PAM gene importance calculation methods is similar to CART. However, in addition to using Sig to calculate importance, PAM used the centroids values (Cen) to calculate gene importance. The detailed calculation method using Cen was as follows. The absolute values were obtained for the averages of the centroids from the PAM analysis results after taking 1,000 repeated samples for the four group pairs (ad/nm, ac/nm, cn/nm and mt/nm). Next, the sum of

the absolute values for each gene was ranked, with 1 point for the lowest value, 2 points for the next lowest value and so on. The same RS was given to identical the centroids values. Finally, the RS of each gene was divided by the highest RS to obtain the RS%.

C5.0

The calculation method based on the number of times each gene being selected, similar to CART, as well as node location. The latter method was that when a gene, as a node, is closer to the root of a decision tree (that is, the first split point), it has a higher RS, while it has a lower RS when it is closer to the tip of the branches and so on. The RS% calculation method is the same as that of CART. The ranked scores were converted to percentiles in order to make it possible to compare the order of gene importance obtained from these four analysis methods.

Table 3 Gene ordering of 55 significant genes via ANN, CART, C50, and PAM methods for the classification of colorectal tumors and normal mucosa tissues

Gene symbol	ANN#			CART∆			C5.0∆			C5.0_importance			PAM∆			PAM_centroid			Sum of RS%	CV
	RI	RS	RS%	Sig	RS	RS%	Sig	RS	RS%	Node	RS	RS%	Sig	RS	RS%	Cen	RS	RS%	(max=6,min=0)	
CA7	5.4	26	0.79	360	18	0.95	307	43	1.00	1.1	28	1.0	100	13	1.00	14.00	13	1.00	5.74	0.09
SPIB	3.2	19	0.58	412	19	1.00	220	39	0.91	1.3	27	1.0	74	7	0.54	7.54	7	0.54	4.52	0.30
GUCA2B	1.8	9	0.27	1	1	0.05	73	25	0.58	2.6	20	0.7	100	13	1.00	14.00	13	1.00	3.62	0.64
AQP8	2.6	15	0.45	0	0	0	74	26	0.60	2.8	17	0.6	95	12	0.92	12.92	12	0.92	3.51	0.59
IL6R	8.1	30	0.91	124	14	0.74	256	41	0.95	1.8	25	0.9	0	0	0	0	0	0	3.49	0.78
SPP1	17.9	33	1.00	185	17	0.89	234	40	0.93	2.9	16	0.6	0	0	0	0	0	0	3.40	0.82
TCN1	7.3	29	0.88	174	15	0.79	279	42	0.98	2.7	19	0.7	0	0	0	0	0	0	3.32	0.79
CWH43	9.4	31	0.94	52	13	0.68	141	36	0.84	2.7	19	0.7	0	0	0	0	0	0	3.14	0.80
SST	2.1	12	0.36	1	1	0.05	80	28	0.65	2.8	18	0.6	76	8	0.62	8.62	8	0.62	2.94	0.49
KIAA1199	7.3	29	0.88	13	8	0.42	118	33	0.77	2.6	20	0.7	0	0	0	0	0	0	2.78	0.84
SLC4A4	1.8	9	0.27	3	3	0.16	34	18	0.42	3.1	14	0.5	79	9	0.69	9.69	9	0.69	2.73	0.48
CHP2	1.1	2	0.06	181	16	0.84	143	37	0.86	1.3	27	1.0	0	0	0	0	0	0	2.73	1.05
GCG	3.2	19	0.58	0	0	0	15	11	0.26	3.7	9	0.3	82	10	0.77	10.77	10	0.77	2.69	0.69
NR3C2	2.9	17	0.52	37	11	0.58	113	32	0.74	2.2	23	0.8	0	0	0	0	0	0	2.66	0.81
NFE2L3	3.0	18	0.55	39	12	0.63	208	38	0.88	3.0	15	0.5	0	0	0	0	0	0	2.60	0.83
CLDN1	11.9	32	0.97	9	7	0.37	103	30	0.70	3.2	13	0.5	0	0	0	0	0	0	2.50	0.92
C9orf125	6.2	28	0.85	7	6	0.32	139	35	0.81	3.1	14	0.5	0	0	0	0	0	0	2.48	0.91
CPM	4.0	22	0.67	6	5	0.26	81	29	0.67	2.4	22	0.8	0	0	0	0	0	0	2.39	0.89
CLDN8	1.5	6	0.18	0	0	0	22	14	0.33	4.1	5	0.2	90	11	0.85	11.85	11	0.85	2.38	0.92
MMP7	5.0	24	0.73	33	10	0.53	76	27	0.63	3.3	12	0.4	0	0	0	0	0	0	2.31	0.82
EDN3	3.7	21	0.64	4	4	0.21	53	22	0.51	2.0	24	0.9	0	0	0	0	0	0	2.22	0.96
GUCA2A	1.2	3	0.09	15	9	0.47	66	24	0.56	1.7	26	0.9	1	1	0.08	1.08	1	0.08	2.21	0.95
CDH3	5.6	27	0.82	1	1	0.05	76	27	0.63	3.1	14	0.5	0	0	0	0	0	0	2.00	1.08
FAM55D	2.0	11	0.33	15	9	0.47	39	20	0.47	2.6	20	0.7	0	0	0	0	0	0	1.99	0.86
NR3C1	1.7	8	0.24	15	9	0.47	106	31	0.72	3.4	11	0.4	0	0	0	0	0	0	1.83	0.93
PYY	2.9	17	0.52	0	0	0	14	10	0.23	3.9	7	0.3	15	5	0.38	5.38	5	0.38	1.77	0.60
SLC30A10	3.7	21	0.64	4	4	0.21	10	6	0.14	4.2	4	0.1	9	4	0.31	4.31	4	0.31	1.74	0.64
BEST2	1.7	8	0.24	0	0	0	132	34	0.79	3.4	11	0.4	2	2	0.15	2.15	2	0.15	1.73	0.96
CLCA1	2.8	16	0.48	15	9	0.47	43	21	0.49	3.9	7	0.3	0	0	0	0	0	0	1.70	0.84
TRPM6	1.6	7	0.21	0	0	0	62	23	0.53	2.4	22	0.8	1	1	0.08	1.08	1	0.08	1.69	1.11
SCNN1B	2.5	14	0.42	2	2	0.11	23	15	0.35	3.9	7	0.3	6	3	0.23	3.23	3	0.23	1.59	0.42
THRB	5.6	27	0.82	0	0	0	33	17	0.40	3.7	9	0.3	0	0	0	0	0	0	1.53	1.28
ABCG2	1.3	4	0.12	0	0	0	34	18	0.42	4.6	2	0.1	43	6	0.46	6.46	6	0.46	1.53	0.84
SPINK5	2.4	13	0.39	1	1	0.05	66	24	0.56	3.2	13	0.5	0	0	0	0	0	0	1.47	1.04
GALNT6	5.2	25	0.76	0	0	0	21	13	0.30	3.6	10	0.4	0	0	0	0	0	0	1.42	1.28
CHST5	1.4	5	0.15	0	0	0	36	19	0.44	2.5	21	0.8	0	0	0	0	0	0	1.34	1.38

Gene symbol	ANN#			CART∆			C5.0∆			C5.0_importance			PAM∆			PAM_centroid			Sum of RS%	CV
	RI	RS	RS%	Sig	RS	RS%	Sig	RS	RS%	Node	RS	RS%	Sig	RS	RS%	Cen	RS	RS%	(max=6,min=0)	
NUP153	4.4	23	0.70	0	0	0	20	12	0.28	3.8	8	0.3	0	0	0	0	0	0	1.26	1.31
DEFA6	3.5	20	0.61	0	0	0	12	8	0.19	4.0	6	0.2	0	0	0	0	0	0	1.01	1.41
SLC7A5	4.4	23	0.70	0	0	0	5	3	0.07	4.0	6	0.2	0	0	0	0	0	0	0.98	1.68
ZG16	1.3	4	0.12	0	0	0	5	3	0.07	2.4	22	0.8	0	0	0	0	0	0	0.98	1.90
C6orf105	1.9	10	0.30	0	0	0	25	16	0.37	3.9	7	0.3	0	0	0	0	0	0	0.93	1.12
HP	3.7	21	0.64	0	0	0	10	6	0.14	4.4	3	0.1	0	0	0	0	0	0	0.88	1.68
MUC2	1.4	5	0.15	2	2	0.11	13	9	0.21	3.6	10	0.4	0	0	0	0	0	0	0.82	0.99
KLK11	2.4	13	0.39	0	0	0	5	3	0.07	3.6	10	0.4	0	0	0	0	0	0	0.82	1.37
AHCYL2	1.4	5	0.15	0	0	0	14	10	0.23	3.3	12	0.4	0	0	0	0	0	0	0.81	1.28
CA1	1.6	7	0.21	0	0	0	9	5	0.12	3.3	12	0.4	0	0	0	0	0	0	0.76	1.36
H3F3A	1.5	6	0.18	0	0	0	12	8	0.19	3.8	8	0.3	0	0	0	0	0	0	0.65	1.15
CLCA4	2.1	12	0.36	0	0	0	3	2	0.05	4.0	6	0.2	0	0	0	0	0	0	0.62	1.46
BTNL3	1.5	6	0.18	0	0	0	11	7	0.16	4.1	5	0.2	0	0	0	0	0	0	0.52	1.10
FCGBP	1.3	4	0.12	0	0	0	12	8	0.19	4.0	6	0.2	0	0	0	0	0	0	0.52	1.15
MS4A12	1.4	5	0.15	2	2	0.11	2	1	0.02	4.0	6	0.2	0	0	0	0	0	0	0.49	1.08
CA4	1.5	6	0.18	0	0	0	7	4	0.09	4.1	5	0.2	0	0	0	0	0	0	0.45	1.17
CD177	1.3	4	0.12	0	0	0	3	2	0.05	4.0	6	0.2	0	0	0	0	0	0	0.38	1.38
SLC26A3	1.0	1	0.03	0	0	0	10	6	0.14	4.2	4	0.1	0	0	0	0	0	0	0.31	1.34
MT1M	1.6	7	0.21	0	0	0	2	1	0.02	5.5	1	0.0	0	0	0	0	0	0	0.27	1.84

Relative importance (RI)：the sum of relative importance values in 1,000 bootstrapping rounds；Ranked score (RS)：the ranked score of each gene；The percentile ranked score (RS%)；Sig: the times of a gene selected as a significant genes in 1,000 bootstrapping rounds; Node：the average location of a gene selected as a node in its decision tree in 1,000 bootstrapping rounds； Centroid (Cen)：the sum of absolute values of centroids for a gene in 1,000 bootstrapping rounds. The higher RS% represents that the gene was more important for the classification of colorectal tumors and normal mucosa tissues. CV stands for coefficient of variance. The RS% of each gene in different methods were calculated by four approaches noted with the suffix, ∆, importance, centroid and #. Each symbol represents the way to calculate RS%. ∆: the significant times of each gene in 1,000 bootstrapping rounds. Importance:The node location of genes in decision trees. Centroid: centroid values of each gene in PAM. #: the relative importance values in ANN.

Table 4 The model performance and the numbers of selected genes of the primary screening of significant genes via PAM in 100 bootstrapping rounds

	ad/nm		ac/nm		cn/nm		mt/nm	
	Mn	Sd	Mn	Sd	Mn	Sd	Mn	Sd
Model accuracy(%)	100.0	1.6	98.6	0.2	95.4	0.5	99.4	0.3
Test accuracy(%)	99.4	0	98.1	1.2	95.1	1.4	98.8	1.9
The average numbers of genes selected	3.9	1.5	6.5	2.6	9.7	2.2	7.4	4.8

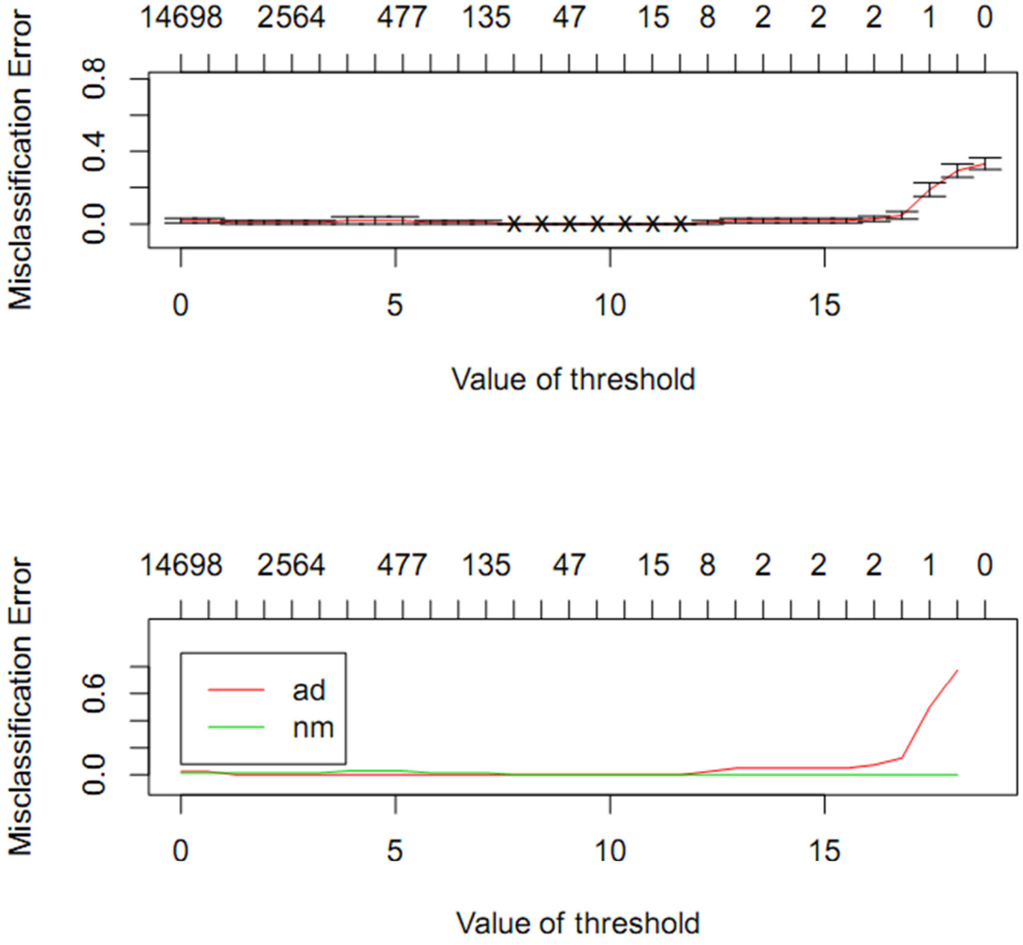
14698 2564 477 135 47 15 8 2 2 2 1 0
Misclassification Error
0.0 0.4 0.8
0 5 10 15
Value of threshold
14698 2564 477 135 47 15 8 2 2 2 1 0
Misclassification Error
0.0 0.6
ad
nm
0 5 10 15
Value of threshold

(a)

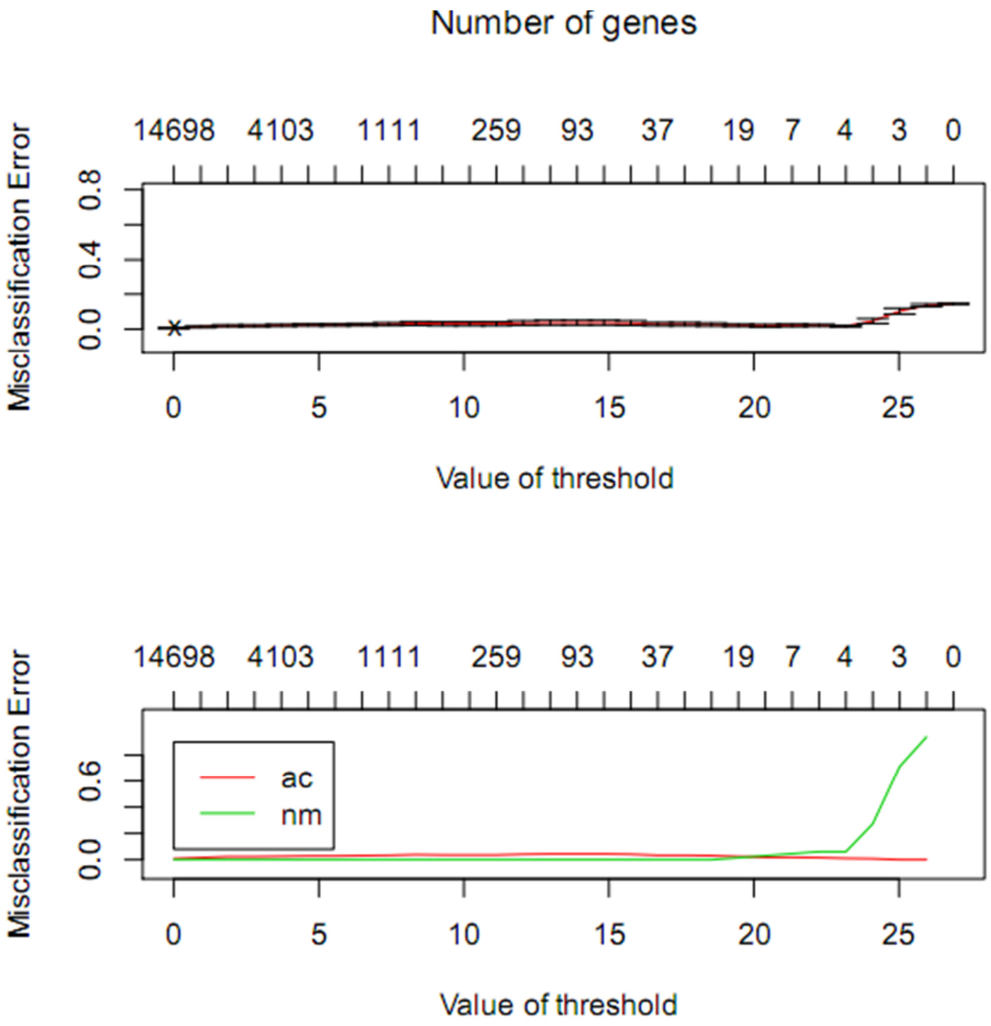
Number of genes
14698 4103 1111 259 93 37 19 7 4 3 0
Misclassification Error
0.0 0.4 0.8
0 5 10 15 20 25
Value of threshold
14698 4103 1111 259 93 37 19 7 4 3 0
Misclassification Error
0.0 0.6
ac
nm
0 5 10 15 20 25
Value of threshold

(b)

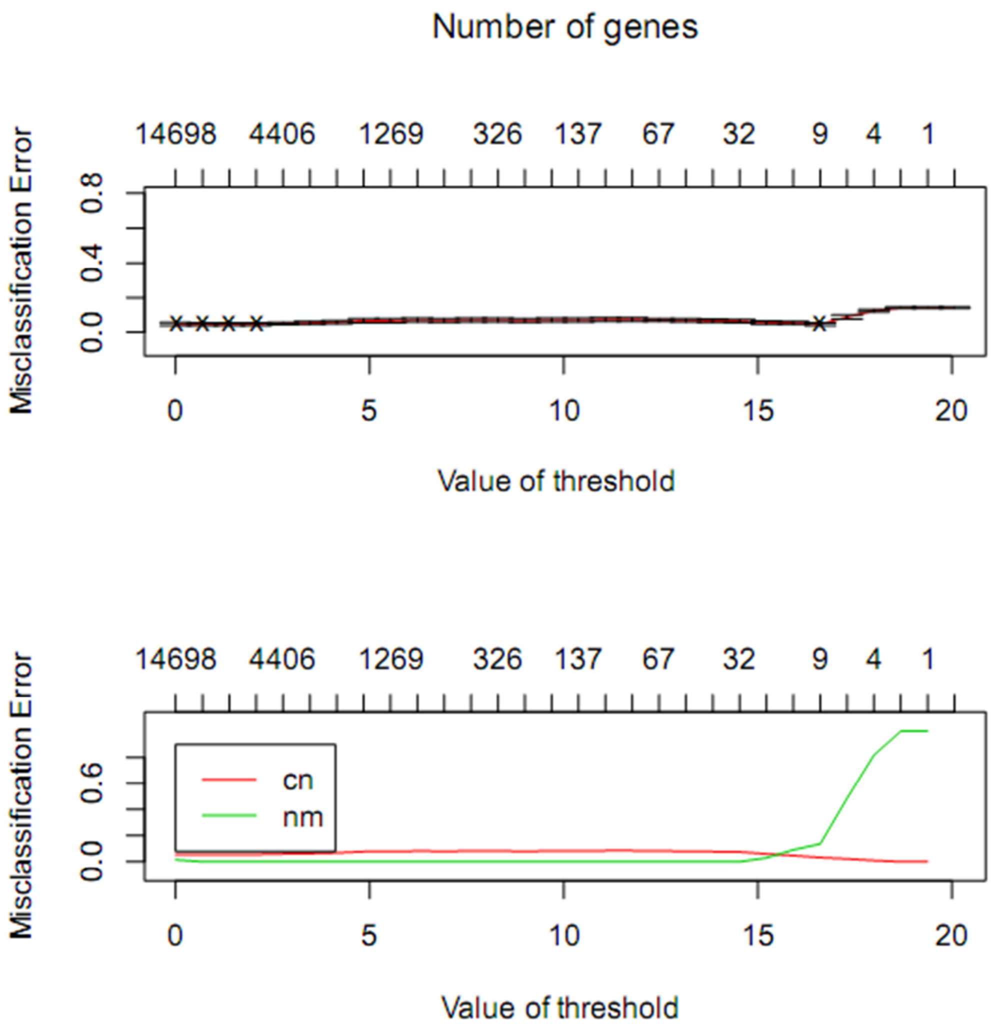

Number of genes
14698
4406
1269
326
137
67
32
9
4
1
Misclassification Error
0.0
0.4
0.8
0
5
10
15
20
Value of threshold
14698
4406
1269
326
137
67
32
9
4
1
Misclassification Error
0.0
0.6
cn
nm
0
5
10
15
20
Value of threshold

(c)

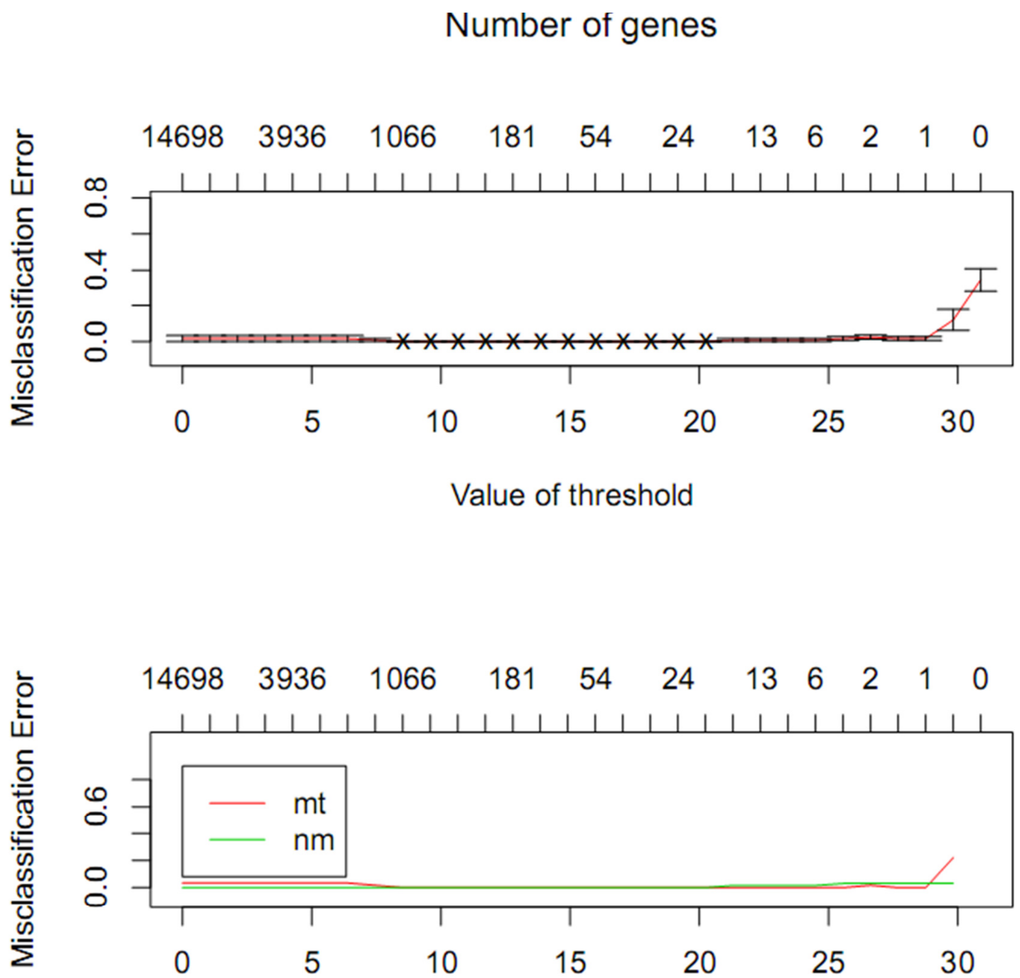
Number of genes
14698 3936 1066 181 54 24 13 6 2 1 0
Misclassification Error
0.0 0.4 0.8
0 5 10 15 20 25 30
Value of threshold
14698 3936 1066 181 54 24 13 6 2 1 0
Misclassification Error
0.0 0.6
mt
nm
0 5 10 15 20 25 30

(d)

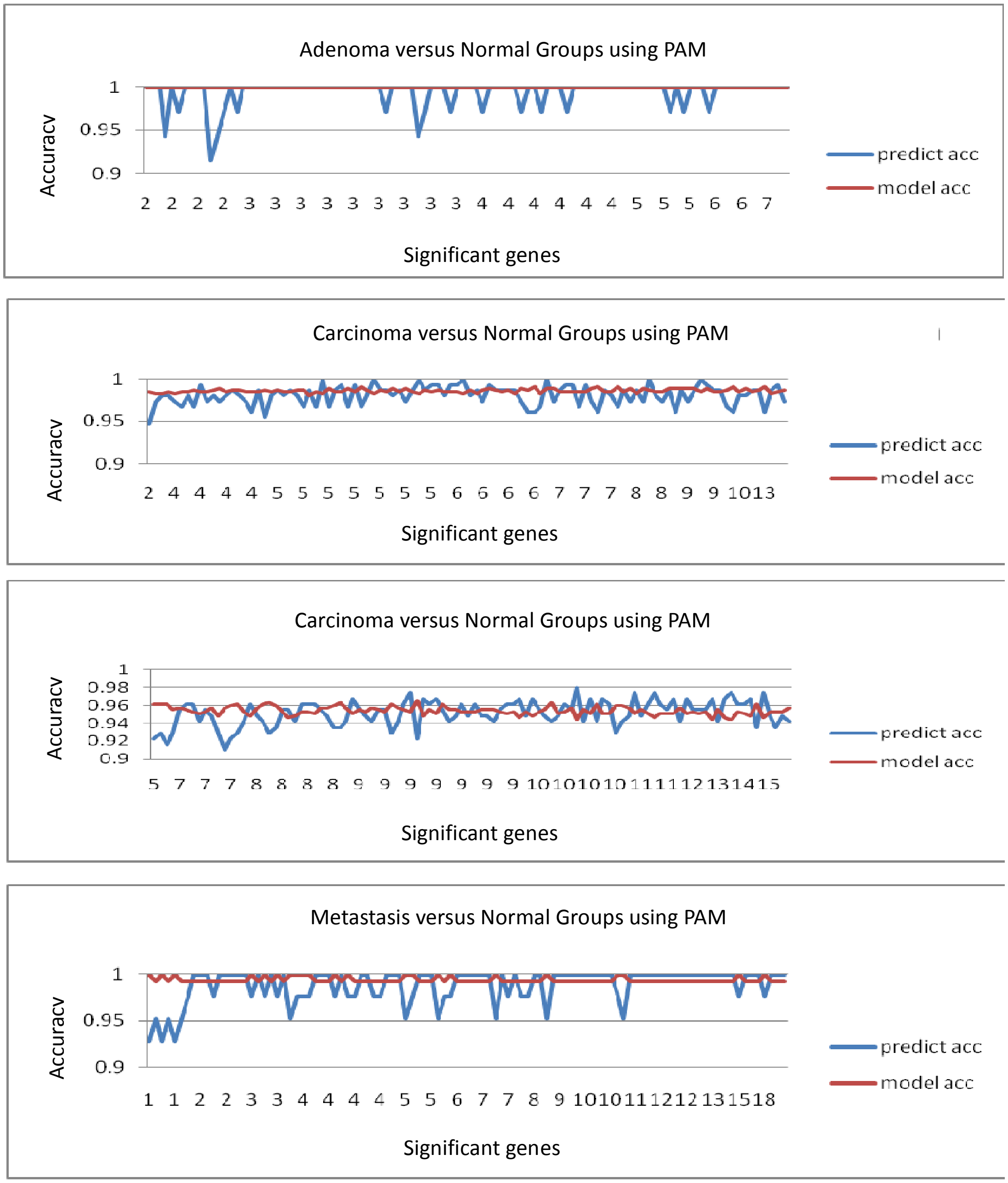

(e)

Figure 1 The figures (a)-(d) showed the relations among misclassification error, numbers of genes and the value of threshold of the PAM methods for the classification of colorectal tumors and normal mucosal tissues. There were four

comparison groups, ad versus nm, ac versus nm, cn versus nm and mt versus nm shown in the figure1(a), (b), (c), and (d), respectively. The (e) shows that PAM screens the candidate genes with highly statistical significances by bootstrapping and cross-validation

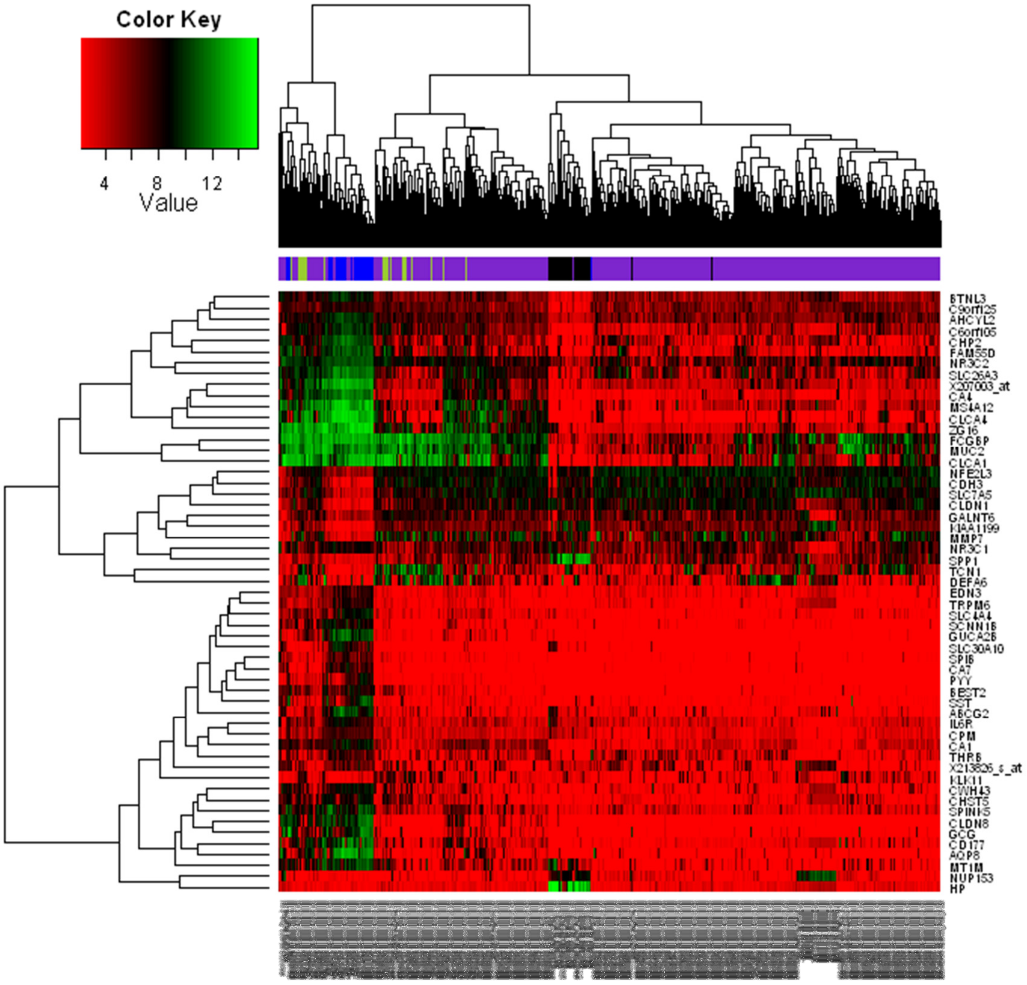

The Heatmap of 1274 samples and 55 PAM primary screening candidate genes using GCRMA log2 normalization, the x axial presents 1274 samples with normal via blue, adenoma via green, adenocarcinoma and carcinoma via purple and metastasis via black. The y axial presents

55 PAM primary screening candidate genes.

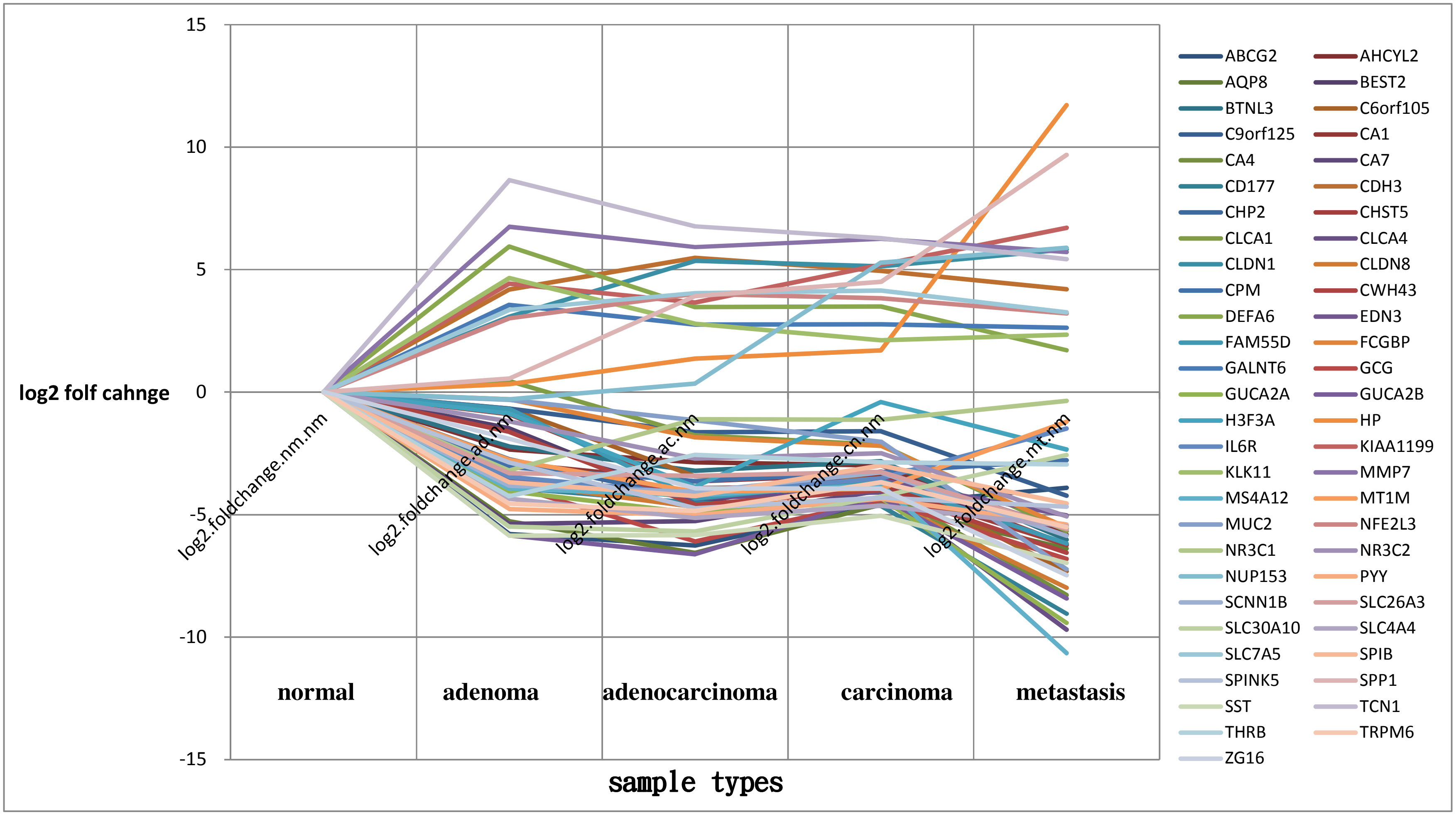

The following is 55 genes via GO molecular function view.

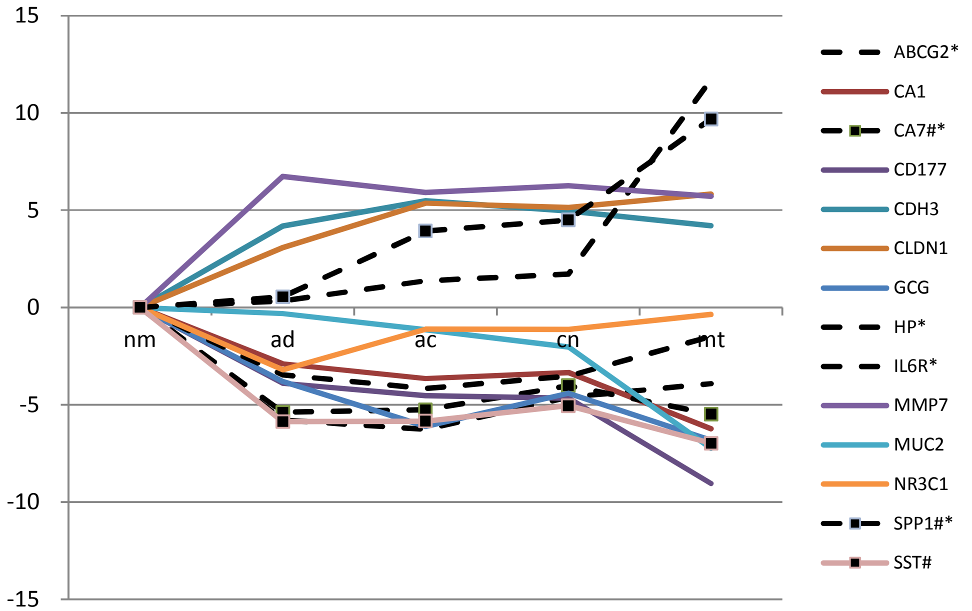

(a) Gelatinase activity

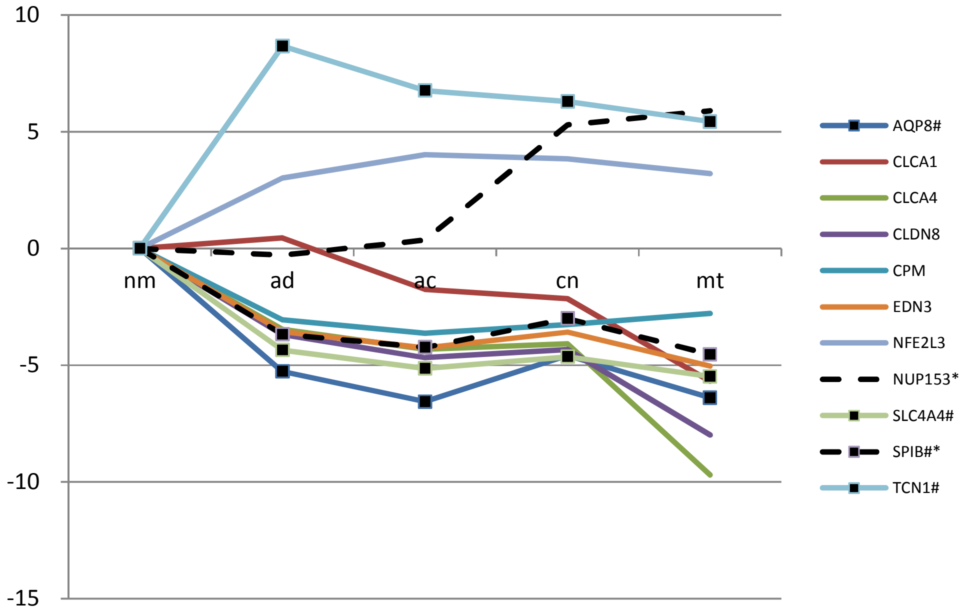

(b) Binding

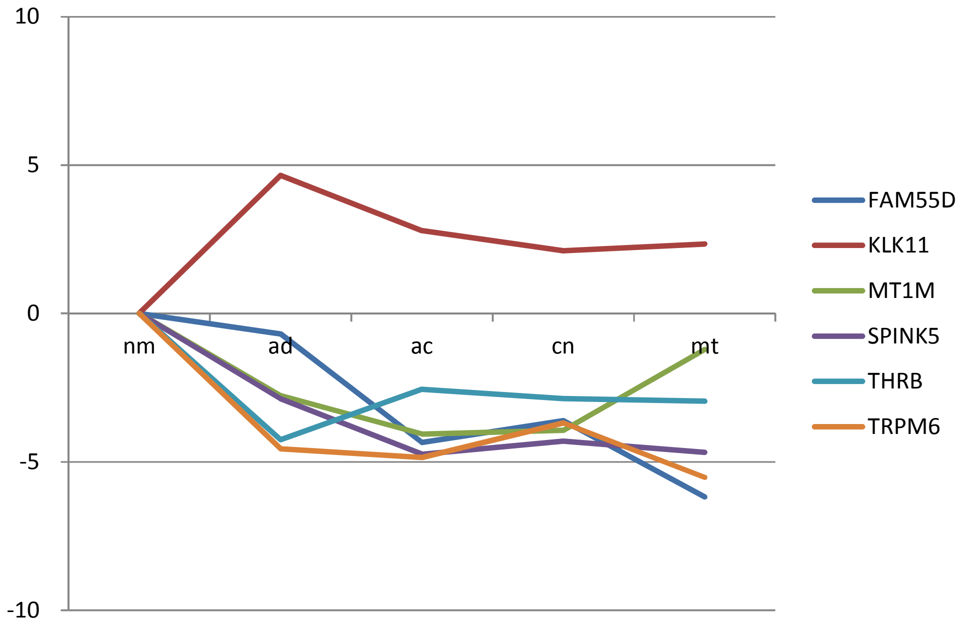

(c) Catalytic activity

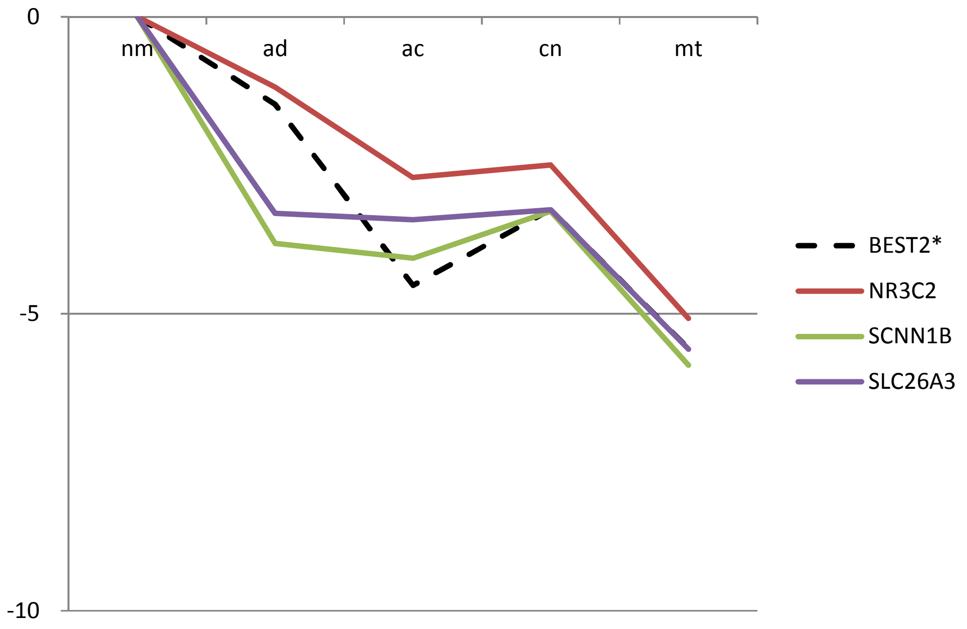

(d) Transporter activity

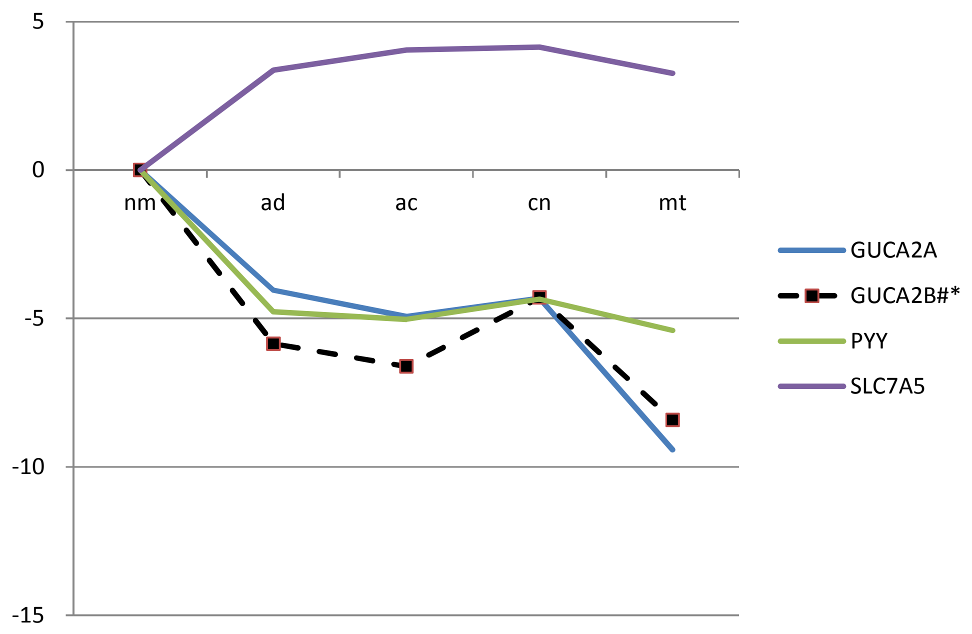

(e) Enzyme regulator activity

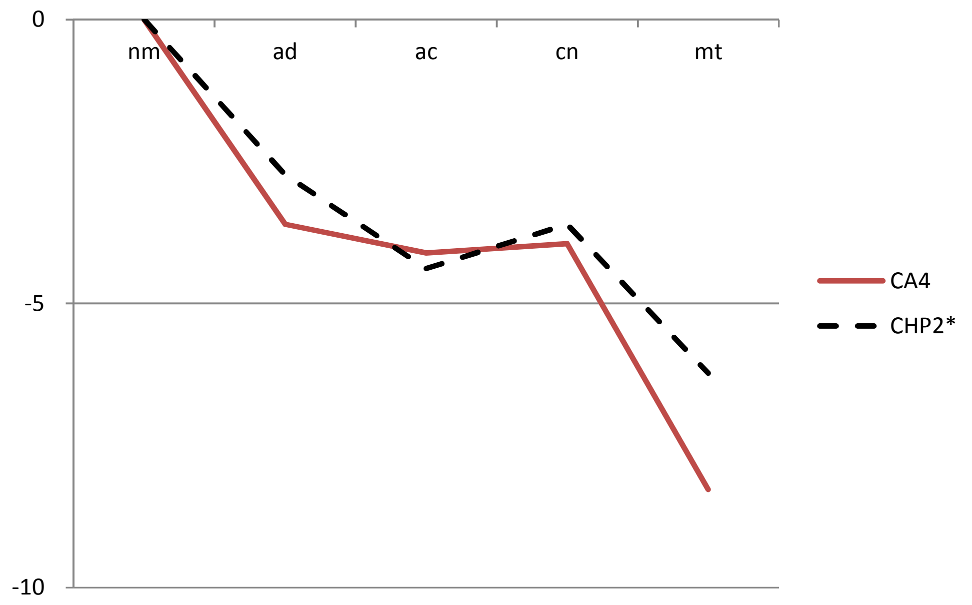

(f) Protein phosphatase type 2B activity

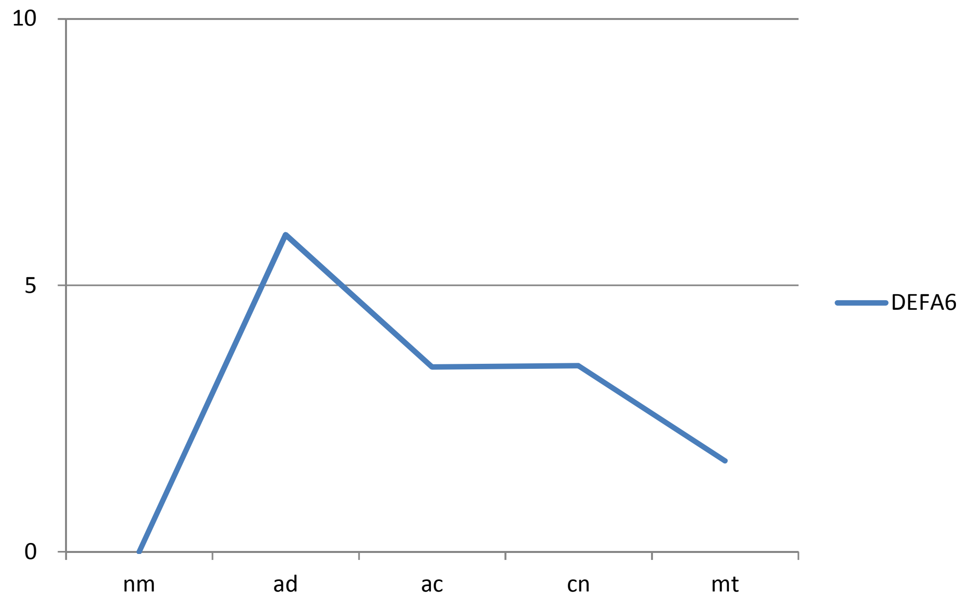

(g) Other-interstitial collagenase activity

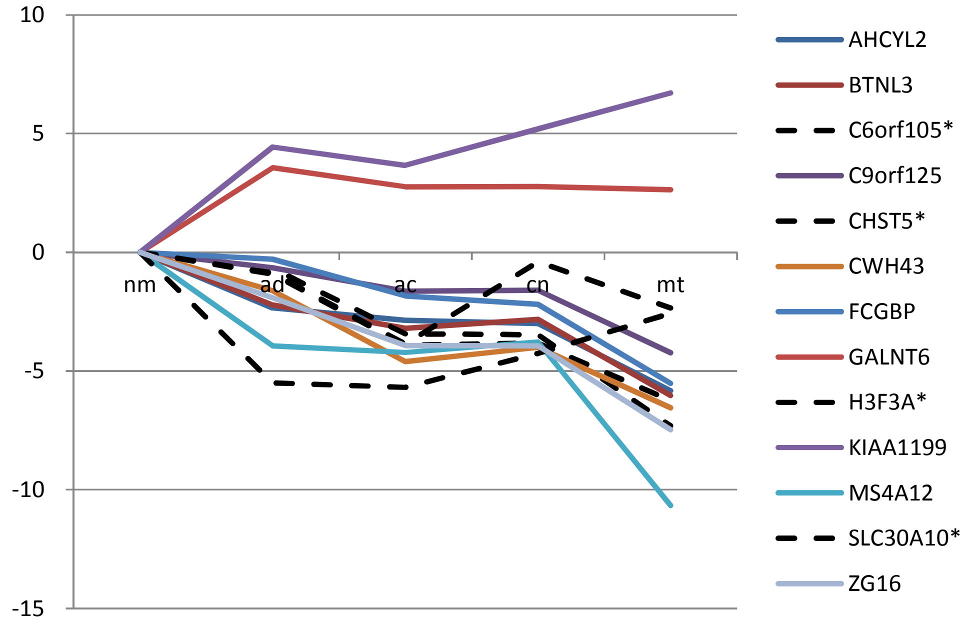

(h) No annotation

The following is 55 genes via GO biology process view.

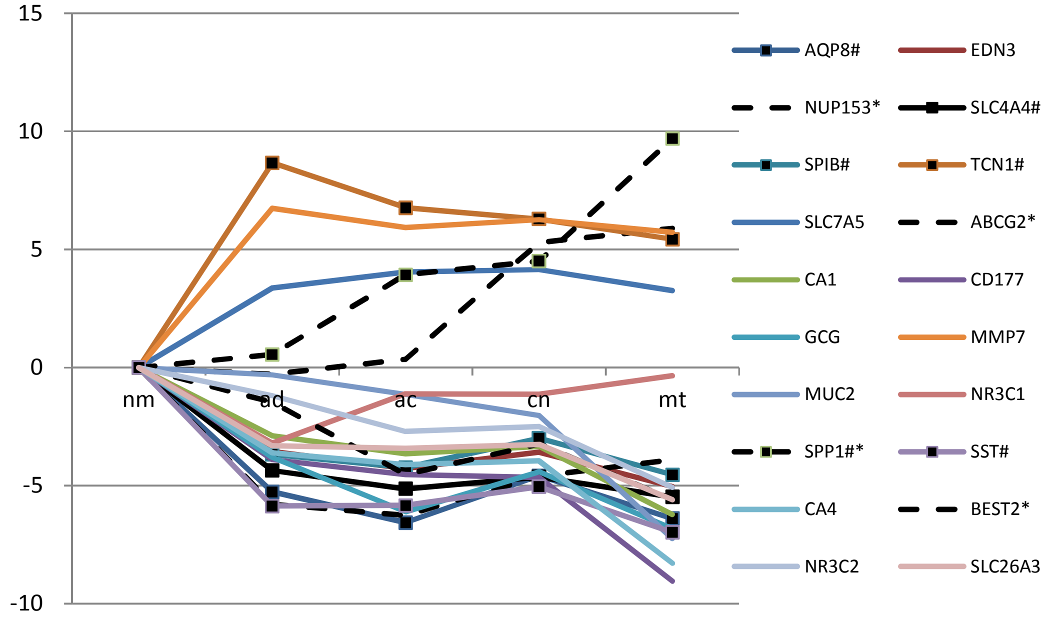

(a) Transporters

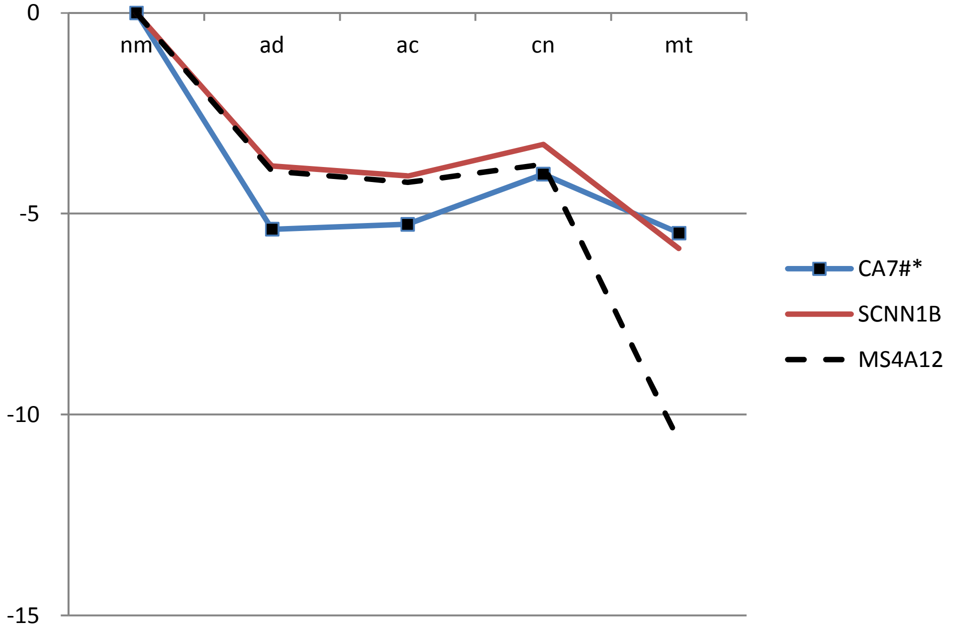

(b) Biological regulation

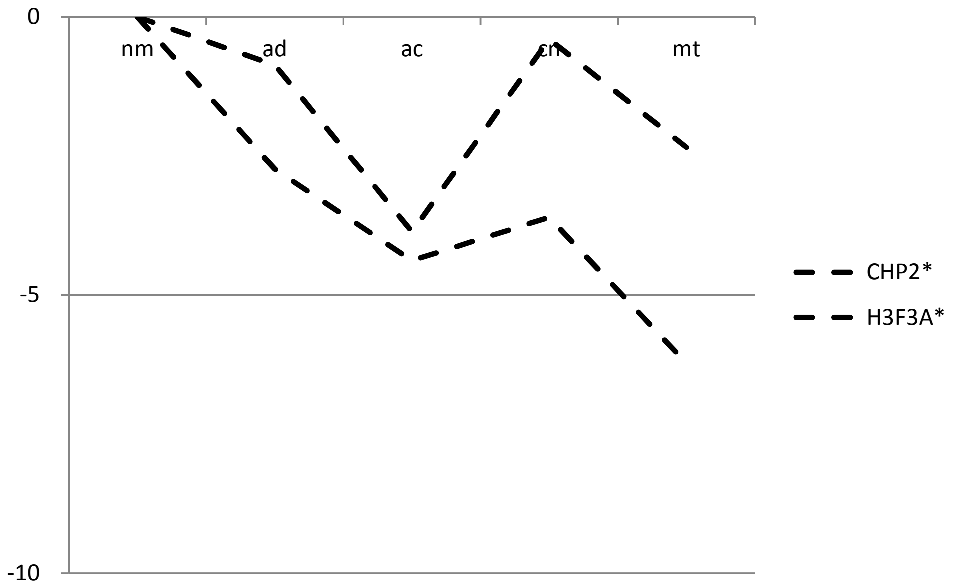

(c) Chromatine modification

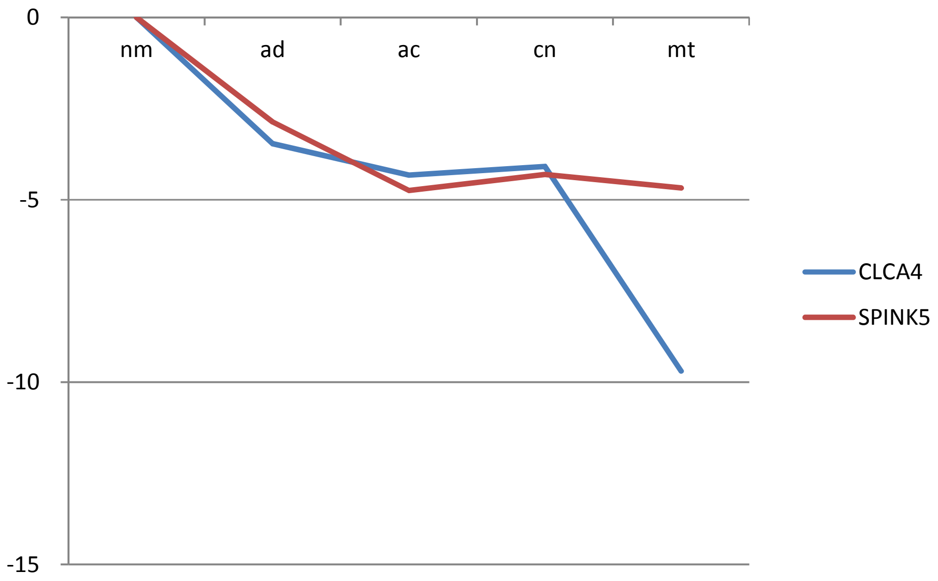

(d) Extracellular matrix organization and biogenesis

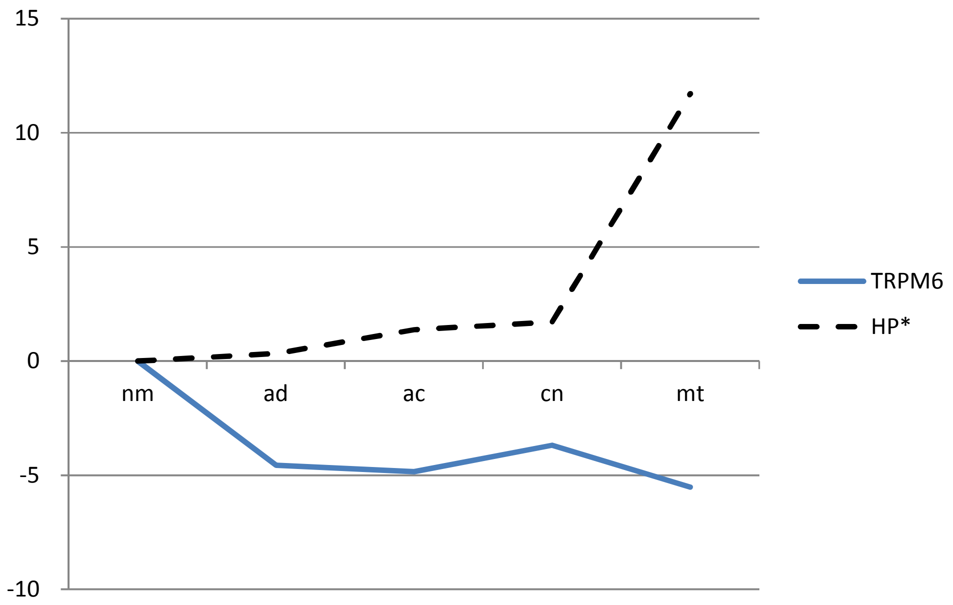

(e) Localization 及 biological regulation

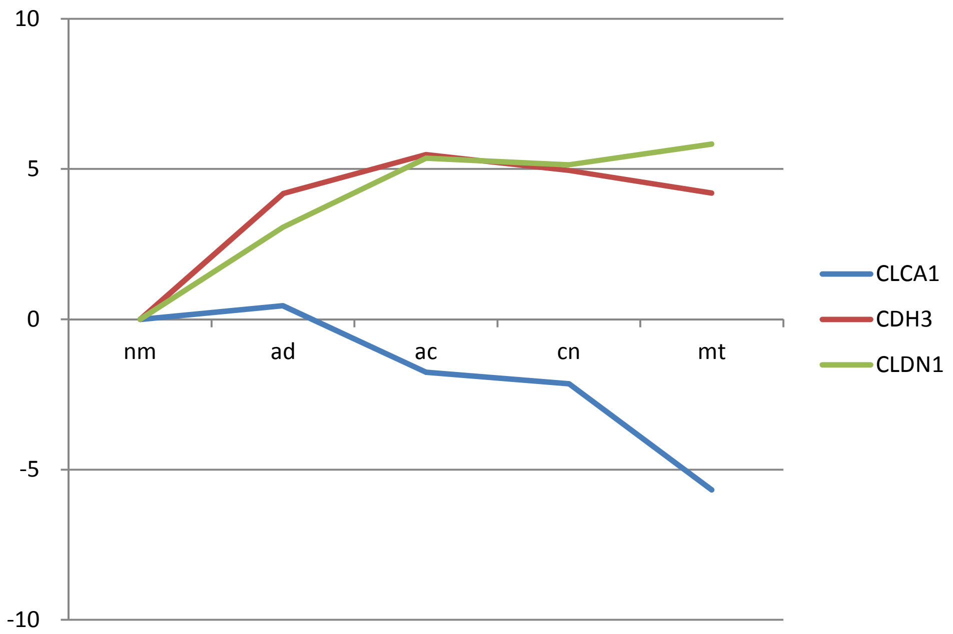

(f) Biological adhesion

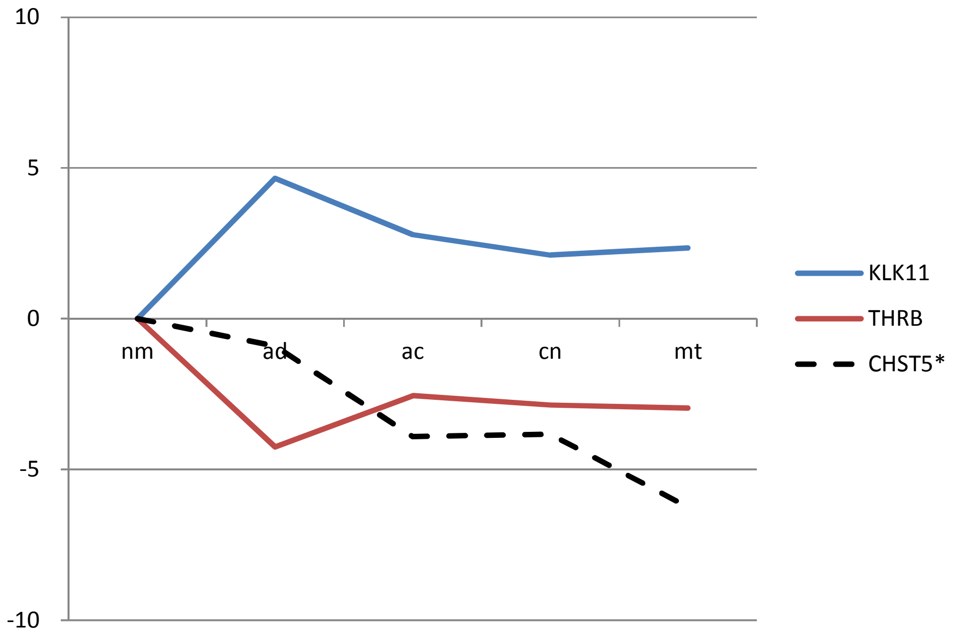

(g) Metabolic process

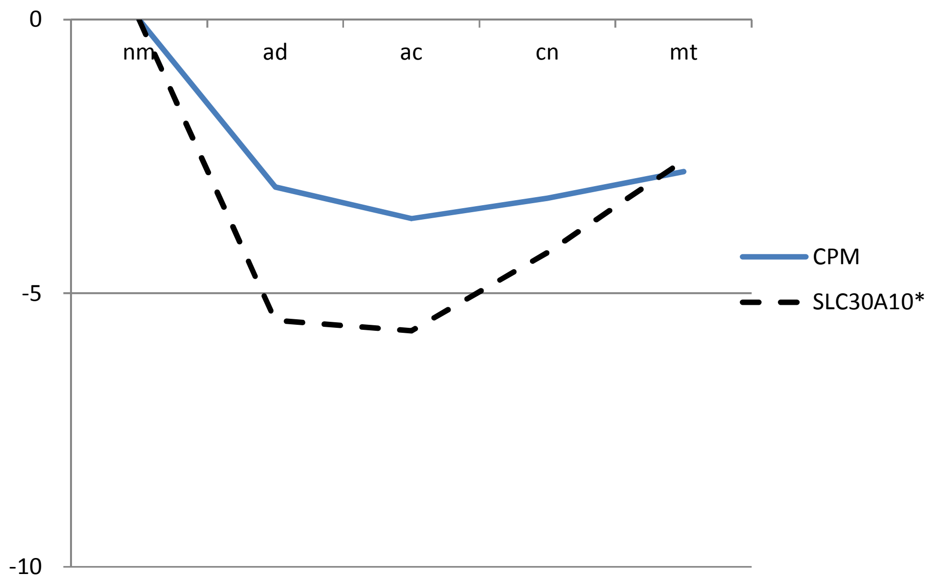

(h) Insulin secretion

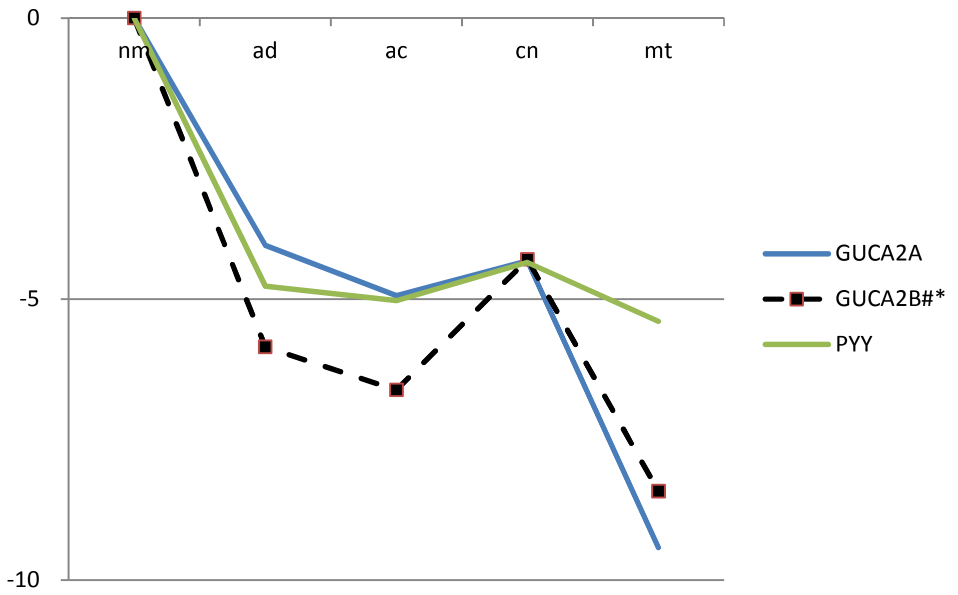

(i) Signaling

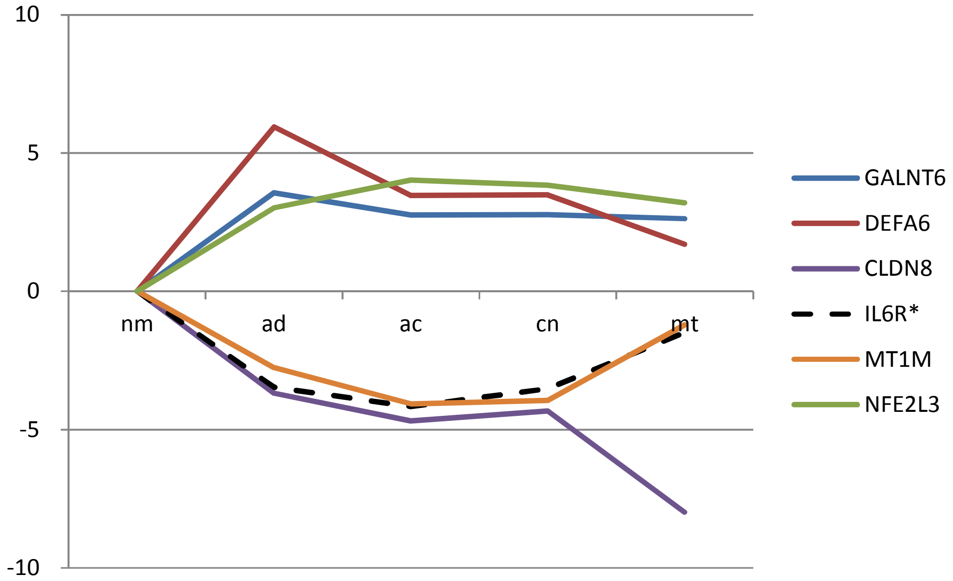

**Genes**	**GO biological process**
GALNT6	cell differentiation and epithelium development
DEFA6	cellular component organization or biogenesis
NFE2L3	developemental process、multicellular organismal process
CLDN8	follicle development
IL6R	immune system process

MT1M	response to stimulus

(j) Others

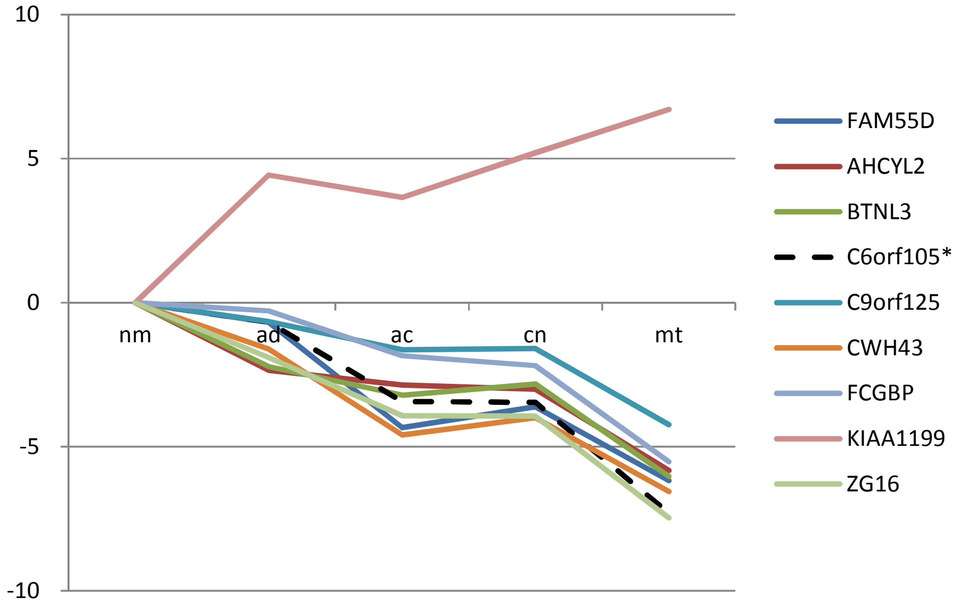

(k) No annotations

Table 5 Gene Ontology **Cellular Component annotations** of 55 significant genes

Gene symbol	Gene Ontology ID	Cellular component	*p
CA7	GO:0009986	cell surface	0
CD177			
CHP2			
CLCA1			
CPM			
DEFA6			
GUCA2A			
GUCA2B			
IL6R			
MS4A12			
NR3C2			
SCNN1B			
SLC26A3			
SLC7A5			
SPIB			
TCN1			
ABCG2	GO:0005923	tight junction	0
AQP8			
CA1			
CDH3			
CLDN1			
CLDN8			
GCG			
HP			
MMP7			
MUC2			
NR3C1			
PYY			
SPP1			
SST			
TRPM6			
SPINK5	GO:0042599	lamellar body	1.94E-06
H3F3A	GO:0016363	nuclear matrix	5.78E-05
FCGBP	GO:0042597	periplasmic space	0.01516
SLC4A4	GO:0016323	basolateral plasma membrane	4.59E-25
CA4	GO:0046658	anchored to plasma	1.98E-14

**Gene symbol**	**Gene Ontology ID**	**Cellular component**	***p**
		membrane	
CLCA4	GO:0016324	apical plasma membrane	1.20E-08
THRB	GO:0033391	chromatoid body	
BEST2	GO:0046930	pore complex	2.32E-05
NUP153	GO:0005643	nuclear pore	0

*The p values of the enrichment analysis. The genes without Gene Ontology Cellular Component annotations were not shown in this table.

Table 6 Gene Ontology **Molecular Function** annotations of 55 significant genes

Gene symbol	Gene Ontology ID	Molecular function	*p
Transporter activity			
SCNN1B	GO:0015280	amiloride-sensitive sodium channel activity	0
SLC26A3			
NR3C2			
BEST2	GO:0015267	channel activity	2.05E-07
Binding			
TCN1	GO:0031419	cobalamin binding	5.79E-165
SLC4A4	GO:0042980	cystic fibrosis transmembrane conductance regulator binding	3.76E-77
NFE2L3	GO:0031995	insulin-like growth factor II binding	0.005004
NUP153	GO:0005521	lamin binding	9.32E-53
AQP8	GO:0042980	cystic fibrosis transmembrane conductance regulator binding	3.76E-77
CLCA1			
CLCA4			
CPM	GO:0005179	hormone activity	2.62E-08
CLDN8	GO:0019864	IgG binding	3.76E-77
SPIB	GO:0004912	interleukin-3 receptor activity	4.62E-10
EDN3	GO:0005102	receptor binding	9.80E-05
Catalytic activity			
TRPM6	GO:0004686	eukaryotic elongation factor-2 kinase activity	6.85E-52
THRB	GO:0004413	homoserine kinase activity	2.28E-247
FAM55D	GO:0016757	transferase activity, transferring glycosyl groups	3.75E-39
SPINK5	GO:0008236	serine-type peptidase activity	1.24E-124
KLK11			
MT1M	GO:0045289	luciferin monooxygenase activity	0.04519
CA4	GO:0004647	protein phosphatase type 2B activity	7.68E-18
CHP2			
Enzyme regulator activity			
PYY	GO:0030250	guanylate cyclase activator activity	0
GUCA2A			
GUCA2B			

Gene symbol	Gene Ontology ID	Molecular function	*p
SLC7A5	GO:0030414	protease inhibitor activity	2.44E-08
Gelatinase activity			
ABCG2	GO:0004229	gelatinase B activity	0
CA1			
CA7			
CD177			
CDH3			
CLDN1			
GCG			
HP			
IL6R			
MMP7			
MUC2			
NR3C1			
SPP1			
SST			
Others			
DEFA6	GO:0004232	interstitial collagenase activity	0

*The p values of the enrichment analysis. The genes without Gene Ontology Molecular Function annotations were not shown in this table.

Table 7 Gene Ontology Biological Process annotations of 55 significant genes

Gene symbol	Gene Ontology ID	Biological process	Categories	*p
Biological adhesion				
CDH3	GO:0016337	cell-cell adhesion	biological adhesion	3.94E-141
CLCA1	GO:0016337			
CLDN1	GO:0016337			
Signaling				
GUCA2A	GO:0007168	receptor guanylyl cyclase signaling pathway	signaling	0
GUCA2B	GO:0007168			
PYY	GO:0007268	synaptic transmission		3.01E-219
Transportation				
ABCG2	GO:0006865	amino acid transport	transport	1.64E-245
CA1	GO:0006865			
CA4	GO:0006865			
CD177	GO:0006865			

Gene symbol	Gene Ontology ID	Biological process	Categories	*p
GCG	GO:0006865			
NR3C1	GO:0030198			
SLC7A5	GO:0030147			
SST	GO:0006865			
AQP8	GO:0006833	water transport		1.13E-135
EDN3	GO:0006833			
SLC26A3	GO:0006833			
SLC4A4	GO:0006833			
MMP7	GO:0042640	nuclear import		2.16E-174
MUC2	GO:0030198			
NUP153	GO:0051170			
SPIB	GO:0030147			
SPP1	GO:0006814			
BEST2	GO:0006811	ion transport		5.71E-13
NR3C2	GO:0046847	sodium ion transport		1.36E-07
TCN1	GO:0035146	cobalamin transport		1.20E-57
Others				
CHP2	GO:0051567	histone H3-K9 methylation	chromatine modification	1.05E-163
H3F3A	GO:0043486	histone exchange		2.66E-38
GALNT6	GO:0031424	keratinization	cell differentiation and epithelium development	1.13E-11

Gene symbol	Gene Ontology ID	Biological process	Categories	*p
DEFA6	GO:0046847	filopodium formation	cellular component organization or biogenesis	1.13E-11
CLCA4 ***SPINK5***	GO:0030198	extracellular matrix organization and biogenesis	extracellular matrix organization and biogenesis	0
NFE2L3	GO:0035146	tube fusion	developemental process, multicellular organismal process	9.96E-112
MS4A12	GO:0016458	gene silencing	biological regulation, metabolic process, cellular process	4.45E-09
CLDN8	GO:0042640	anagen	follicle development	1.95E-07
IL6R	GO:0030225	macrophage differentiation	immune system process	3.05E-15
KLK11	GO:0031638	zymogen activation	metabolic process	1.47E-07
CHST5	GO:0018146	keratan sulfate biosynthetic process	metabolic process, cellular process	7.03E-199
THRB	GO:0009088	threonine biosynthetic process	metabolic process, cellular process	0
MT1M	GO:0006805	xenobiotic metabolic process	response to stimulus	8.15E-18
CPM ***SLC30A10***	GO:0030073	insulin secretion	insulin secretion	6.54E-76
HP ***TRPM6***	GO:0030147	natriuresis	localization, biological regulation	6.55E-26
CA7	GO:0055081	anion homeostasis	biological regulation	2.00E-297
SCNN1B	GO:0030104	water homeostasis		1.67E-76

*The p values of the enrichment analysis. The genes without Gene Ontology Biological Process annotations were not shown in this table.

Table 8 The numbers of selected genes using PAM, CART, C5.0 and ANN in 1,000 bootstrapping rounds for classifying colorectal tumors and normal mucosal tissues.

Gene selection model	min	median	Max	Mean
PAM	2	8	11	7.7
CART	1	2	4	1.7
C5.0	1	4	7	3.7
ANN	5	5	5	5

ANN can not filter out the significant genes as do the other three methods. Therefore, we split the total of 55 input variables (genes) into 5,10,15,…,55 genes on the basis of the relative improtance of genes. This table presented the number of genes with the relative good performance of the ANN model.

Table 9 Multivariate logistic regression of 55significant genes and demographics

Gene symbol	Model1[#] (n=1,274)		Model2[&] (n=759)		Model3[||] (n=1,274)		Model4[†] (n=272)		Model5[%] (n=722)		Model6[@] (n=185)	
	OR	p	OR	p	OR	p	OR	p	OR	p	OR	p
ABCG2	0.47	***	0.18	***	0.47	***	0.55	***	0.18	***	0.19	*
AHCYL2	0.14	***	4.74E-3	**	0.14	***	0.22	***	0.01	**	0.02	*
AQP8	0.57	***	0.27	***	0.58	***	0.62	***	0.27	***	0.17	0.06
BEST2	0.35	***	0.38	***	0.30	***	0.31	***	0.40	***	0.51	*
BTNL3	0.20	***	0.19	***	0.21	***	0.24	***	0.21	***	0.21	*
C6orf105	0.32	***	0.20	***	0.30	***	0.35	***	0.23	***	0.04	0.06
C9orf125	0.05	***	0.06	***	0.05	***	0.06	***	0.06	***	0.04	**
CA1	0.24	***	0.22	***	0.25	***	0.27	***	0.22	***	0.17	0.07
CA4	0.41	***	0.21	***	0.43	***	0.47	***	0.28	***	0.33	0.10
CA7	0.29	***	0.30	***	0.29	***	0.38	***	0.34	***	0.46	**
CD177	0.53	***	0.37	***	0.52	***	0.53	***	0.40	***	0.36	*
CDH3	3.08	***	3.13	***	3.03	***	3.32	***	2.98	***	3.01	0.22
CHP2	0.21	***	0.24	***	0.19	***	0.25	***	0.23	***	2.14E-4	*
CHST5	0.37	***	0.48	***	0.33	***	0.26	***	0.51	***	0.42	*
CLCA1	0.62	***	0.70	***	0.53	***	0.60	***	0.71	***	0.63	*
CLCA4	0.48	***	0.09	***	0.51	***	0.61	***	0.09	***	0.04	0.18
CLDN1	6.16	***	7.28	***	5.25	***	5.00	***	6.49	***	3.54	0.53
CLDN8	0.45	***	0.46	***	0.44	***	0.39	***	0.47	***	0.65	*
CPM	0.20	***	0.23	***	0.20	***	0.22	***	0.25	***	0.29	*
CWH43	0.21	***	0.04	***	0.20	***	0.13	***	0.04	***	0.07	*
DEFA6	1.34	***	1.73	**	1.38	***	1.35	***	1.75	**	1.52	0.18
EDN3	0.19	***	0.18	***	0.19	***	0.22	***	0.18	***	0.27	*
FAM55D	0.29	***	0.11	***	0.29	***	0.28	***	0.12	***	0.20	0.05
FCGBP	0.35	***	0.41	***	0.29	***	0.26	***	0.39	***	0.24	*
GALNT6	2.06	***	2.00	***	2.33	***	1.43	***	1.95	***	2.31	*
GCG	0.52	***	0.59	***	0.44	***	0.53	***	0.60	***	0.59	*
GUCA2A	0.41	***	0.23	***	0.42	***	0.50	***	0.25	***	0.33	0.09
GUCA2B	0.47	***	0.37	***	0.48	***	0.57	***	0.39	***	0.41	0.11
H3F3A	0.63	***	0.62	***	0.49	***	0.99	0.9	0.60	***	0.42	*
HP	2.83	***	32.99	***	1.65	0.06	4.46	***	30.50	***	2.86E+11	*
IL6R	0.24	***	0.22	***	0.20	***	0.27	***	0.19	***	0.05	*
KIAA1199	11.43	***	22.93	***	11.63	***	6.52	***	84.47	**	4.95	0.13
KLK11	2.16	***	4.09E+6	***	1.70	***	1.69	**	1.79E+6	**	3.57E+9	0.10
MMP7	3.31	***	6.86	***	3.30	***	4.00	***	5.59	***	-	-
MS4A12	0.39	***	0.20	***	0.42	***	0.46	***	0.22	***	0.33	0.09

Gene symbol	Model1[♯] (n=1,274)		Model2[&] (n=759)		Model3[‖] (n=1,274)		Model4[+] (n=272)		Model5[%] (n=722)		Model6[@] (n=185)	
	OR	p	OR	p	OR	p	OR	p	OR	p	OR	p
MT1M	0.48	***	0.43	***	0.44	***	0.40	***	0.46	***	0.70	*
MUC2	0.58	***	0.68	***	0.49	***	0.48	***	0.66	**	0.63	0.07
NFE2L3	2.73	***	2.57	***	2.82	***	2.57	***	2.39	***	1.55	0.05
NR3C1	0.45	***	0.40	***	0.44	***	0.36	***	0.48	***	0.78	0.20
NR3C2	0.05	***	0.07	***	0.05	***	0.07	***	0.09	***	0.01	*
NUP153	1.61	***	1.35	0.2	2.04	**	1.76	***	1.36	0.2	1.24	0.71
PYY	0.31	***	0.34	***	0.27	***	0.27	***	0.39	***	0.62	0.06
SCNN1B	0.41	***	0.43	***	0.42	***	0.38	***	0.46	***	0.65	*
SLC26A3	0.27	***	0.09	***	0.28	***	0.33	***	0.10	***	0.21	0.08
SLC30A10	0.39	***	0.31	***	0.37	***	0.47	***	0.26	***	0.42	*
SLC4A4	0.37	***	0.05	***	0.35	***	0.31	***	0.06	**	0.03	*
SLC7A5	3.38	***	5.93	***	3.29	***	3.59	***	5.72	***	3.51	0.14
SPIB	0.25	***	0.34	***	0.25	***	0.36	***	0.35	***	0.61	*
SPINK5	0.38	***	0.40	***	0.37	***	0.33	***	0.43	***	0.59	*
SPP1	5.55	***	5.47	***	8.67	***	3.46	***	5.85	***	2.24	0.13
SST	0.46	***	0.51	***	0.40	***	0.44	***	0.56	***	0.77	0.15
TCN1	5.16	***	1.09E+07	*	11.70	***	3.29	***	4.03E+3	0.1	4.85E+40	0.48
THRB	0.38	***	0.21	***	0.39	***	0.31	***	0.25	***	0.27	*
TRPM6	0.30	***	0.25	***	0.32	***	0.39	***	0.26	***	0.39	*
ZG16	0.33	***	0.22	***	0.26	***	0.18	***	0.20	***	0.28	*

The p values were from the logistic regression.*<0.05, **<0.01 and ***<0.001

[&]control the variable of genders with males as the reference group

[‖] control the variable of races with the European people as the reference group

[+] control the variable of the location of tissues, with the samples of proximal position as the reference group

[%] control the variable of ages with the people of ≤ 60 years as the reference group

[@]control the variables of gender, races, location of tissues and ages

Table 10　Multivariate logistic regression of 55 significant genes and clinical variables

Gene symbol	Outcomes (dependent variable)											
	MSI[a] (n=90)		Stage2[b] (n=196)		Stage3[b] (n=221)		Stage4[b] (n=147)		Grade 2[c] (n=231)		Grade 3[c] (n=65)	
	OR	p	OR	p	OR	p	OR	p	OR	p	OR	p
ABCG2	0.83	**	1.05	0.69	1.19	0.10	1.17	0.17	3.20	0.09	3.70	0.06
AHCYL2	1.17	*	0.91	0.39	0.91	0.40	0.89	0.32	0.83	0.30	0.53	**
AQP8	0.88	*	1.02	0.84	1.09	0.28	0.99	0.87	1.88	0.27	1.66	0.38
BEST2	0.78	*	0.92	0.55	0.87	0.32	0.76	0.11	1.33	0.41	1.08	0.84
BTNL3	0.62	***	0.91	0.35	0.96	0.70	0.74	*	1.71	*	2.17	**
C6orf105	0.93	0.18	0.83	*	0.81	**	0.80	*	0.92	0.53	0.66	*
C9orf125	1.28	*	1.10	0.47	0.89	0.37	0.99	0.96	0.87	0.55	1.30	0.29
CA1	0.74	***	1.02	0.84	0.94	0.54	0.88	0.25	1.08	0.65	0.67	*
CA4	0.91	*	0.99	0.92	0.98	0.78	0.91	0.12	1.25	0.15	1.02	0.92
CA7	0.49	*	0.73	0.20	0.84	0.39	0.78	0.32	1.8E+88	***	1.8E+88	-
CD177	0.99	0.85	1.06	0.45	1.10	0.24	0.62	***	1.40	0.21	1.45	0.19
CDH3	1.28	**	1.17	0.12	1.07	0.51	1.03	0.74	0.89	0.57	0.64	*
CHP2	0.80	***	0.89	0.07	0.88	*	0.92	0.20	1.37	*	1.54	**
CHST5	1.19	**	0.85	0.07	0.82	*	0.60	***	0.98	0.90	1.20	0.33
CLCA1	0.96	0.19	0.92	*	0.86	***	0.89	**	0.99	0.87	0.93	0.30
CLCA4	0.96	0.16	1.03	0.42	0.99	0.84	0.99	0.85	1.12	0.22	1.01	0.89
CLDN1	0.97	0.50	1.09	0.24	0.95	0.47	1.16	0.08	1.01	0.91	0.81	0.10
CLDN8	0.76	***	0.97	0.59	0.99	0.83	0.88	0.10	1.37	0.22	1.19	0.52
CPM	0.54	***	1.14	0.45	1.14	0.45	1.12	0.56	1.08	0.81	0.39	*
CWH43	0.72	***	0.89	0.14	0.88	0.11	0.93	0.38	1.17	0.40	1.39	0.08
DEFA6	0.77	***	0.97	0.49	0.85	***	0.95	0.21	0.99	0.85	1.02	0.76
EDN3	0.53	***	0.90	0.51	1.07	0.65	0.77	0.13	1.19	0.58	1.44	0.26
FAM55D	0.71	***	0.95	0.42	0.92	0.20	0.99	0.87	1.08	0.52	0.88	0.41
FCGBP	1.12	**	0.92	0.06	0.86	***	0.86	***	0.87	0.08	0.88	0.15
GALNT6	0.71	***	1.02	0.83	1.14	0.08	1.06	0.43	0.68	**	0.44	***
GCG	0.80	**	0.99	0.94	1.00	0.97	0.96	0.60	1.33	0.23	1.24	0.37
GUCA2A	0.79	***	1.00	0.96	1.04	0.59	0.96	0.60	1.34	0.10	1.08	0.68
GUCA2B	0.87	*	0.94	0.68	0.96	0.76	0.87	0.39	3.42	0.34	2.58	0.47
H3F3A	0.18	***	0.96	0.50	1.05	0.43	1.03	0.65	1.10	0.38	1.28	*
HP	0.55	*	0.98	0.89	1.09	0.54	1.03	0.86	1.97	0.18	3.70	*
IL6R	0.66	***	1.01	0.97	0.98	0.86	1.02	0.86	1.07	0.80	0.92	0.77
KIAA1199	0.91	0.17	1.10	0.27	1.06	0.51	0.92	0.39	0.99	0.95	1.34	*
KLK11	1.26	***	1.06	0.46	1.07	0.41	0.99	0.89	0.83	0.10	0.80	0.10
MMP7	0.84	***	1.15	*	1.20	***	1.12	*	1.07	0.52	0.87	0.20

Gene symbol	Outcomes (dependent variable)											
	MSI[a] (n=90)		Stage2[b] (n=196)		Stage3[b] (n=221)		Stage4[b] (n=147)		Grade 2[c] (n=231)		Grade 3[c] (n=65)	
	OR	p	OR	p	OR	p	OR	p	OR	p	OR	p
MS4A12	0.87	***	1.02	0.74	1.04	0.40	0.99	0.80	1.43	*	1.33	0.10
MT1M	1.09	*	0.98	0.76	1.02	0.70	0.91	0.17	1.13	0.36	0.96	0.77
MUC2	1.15	***	0.96	0.31	0.93	0.09	0.89	**	0.84	*	0.75	**
NFE2L3	1.06	0.42	0.87	0.25	0.87	0.24	0.97	0.83	0.83	0.40	0.62	*
NR3C1	0.96	0.55	1.07	0.32	1.10	0.16	1.24	**	0.92	0.50	0.70	**
NR3C2	1.10	0.10	0.98	0.83	0.83	*	0.96	0.61	1.00	1.00	0.98	0.86
NUP153	0.89	0.10	0.96	0.44	1.06	0.18	0.87	*	1.08	0.35	1.28	**
PYY	0.41	*	1.24	0.49	1.14	0.67	1.13	0.70	1.2E+63	***	7.0E+62	-
SCNN1B	0.73	**	1.00	0.99	1.00	0.97	0.93	0.58	109	0.23	61	0.29
SLC26A3	0.75	***	0.98	0.78	1.03	0.63	1.03	0.69	1.30	*	1.04	0.80
SLC30A10	0.71	*	0.69	0.13	0.64	0.09	0.93	0.69	2.2E+96	***	2.07E+96	-
SLC4A4	0.75	**	1.09	0.48	1.08	0.53	0.72	*	1.19	0.53	0.92	0.79
SLC7A5	1.22	**	1.15	0.18	1.21	0.07	1.03	0.81	1.14	0.43	1.80	**
SPIB	0.62	**	0.83	0.53	1.18	0.50	0.88	0.68	6.5E+05	0.20	7.1E+05	0.20
SPINK5	0.89	*	1.10	0.30	1.13	0.18	1.04	0.66	1.22	0.33	1.20	0.40
SPP1	1.10	0.11	1.38	***	1.44	***	1.50	***	1.04	0.71	0.95	0.68
SST	0.64	**	0.87	0.32	0.83	0.18	0.90	0.44	2.88	0.30	1.89	0.55
TCN1	1.12	**	1.04	0.38	0.98	0.68	0.97	0.59	0.76	***	0.84	*
THRB	0.55	***	1.06	0.53	1.03	0.69	1.24	*	1.12	0.52	0.74	0.19
TRPM6	0.26	***	0.89	0.39	1.01	0.93	0.70	*	1.68	0.12	2.82	**
ZG16	0.91	**	0.97	0.50	0.93	0.08	0.95	0.24	1.13	0.16	1.00	0.98

Dependent variables were clinical variables.

a. Reference group was MSS (n=280).

b. Reference group was Stage1 (n=86).

c. Reference group was Grade1 (n=21).

Table 11 Significant functional pathways in the comparison of colorectal tumors and normal mucosal tissues via the method of Gene Set Enrichment Analysis(GSEA). The input variables were the gene expression of 55 genes.

MSigDB	Names of gene sets	Enriched in*	Category	# of genes	NES	NOM.p.val	FDR.q.val
C1_ALL	CHR7P14	crc		21	-1.71	0	0.23
	CHR7Q32			25	1.72	0	0.22
	CHR13Q12			45	1.69	0.02	0.22
C2_CP	HSA04110_CELL_CYCLE	crc	cell cycle	103	-1.83	0	0.06
	HSA03020_RNA_POLYMERASE		trancription	18	-1.86	0.01	0.07
	HSA00564_GLYCEROPHOSPHOLIPID_ METABOLISM	nm	lipid metabolism	55	1.79	0	0.17
	HSA00565_ETHER_LIPID_METABOLISM			27	1.80	0	0.20
C5_BP	CELL_CYCLE_PROCESS	crc	cell cycle	169	1.81	0.01	0.05
	INTERPHASE			63	1.85	0	0.06
	INTERPHASE_OF_MITOTIC_CELL_CYCLE			57	1.78	0	0.08
	DNA_DEPENDENT_DNA_REPLICATION		DNA replication	53	1.83	0	0.06
	DNA_REPLICATION			97	1.89	0	0.11
	AEROBIC_RESPIRATION	nm	metabolic process	15	-1.91	0	0.03
	DNA_METABOLIC_PROCESS	crc		241	1.81	0	0.06
	FEEDING_BEHAVIOR	nm	response to stimulus	20	-2.10	0	0
	GLUTAMATE_SIGNALING_PATHWAY	crc	signaling	17	1.88	0	0.06
C5_CC	INTRINSIC_TO_ENDOPLASMIC_RETICULUM_ MEMBRANE	nm		20	-1.83	0	0.04

MSigDB	Names of gene sets	Enriched in*	Category	# of genes	NES	NOM.p.val	FDR.q.val
	ENDOPLASMIC_RETICULUM_MEMBRANE			73	-1.70	0	0.08
	INTEGRAL_TO_ENDOPLASMIC_RETICULUM_ MEMBRANE			20	-1.83	0	
	INTRINSIC_TO_ORGANELLE_MEMBRANE			44	-1.77	0	0.05
	INTEGRAL_TO_ORGANELLE_MEMBRANE			42	-1.76	0	0.05
	PROTEASOME_COMPLEX	crc		23	1.71	0.02	0.11
	RIBONUCLEOPROTEIN_COMPLEX			113	1.71	0.01	0.14
	NUCLEOLUS			102	1.80	0	0.18
	NUCLEAR_CHROMOSOME			45	1.71	0	0.18
	CONDENSED_CHROMOSOME			29	1.72	0	0.24
C5_mf	DOUBLE_STRANDED_DNA_BINDING	crc	binding→nucleic acid binding	31	1.81	0	0.16
	STRUCTURE_SPECIFIC_DNA_BINDING		binding→nucleic acid binding	53	1.75	0.01	0.21
	CHROMATIN_BINDING		binding→chromatin binding	29	1.74	0.01	0.15
	NEUROPEPTIDE_BINDING	nm	binding→peptide binding	20	-2.14	0	0
	CHANNEL_REGULATOR_ACTIVITY		channel regulator activity	22	-1.79	0.01	0.02
	LIGAND_DEPENDENT_NUCLEAR_RECEPTOR_ ACTIVITY		signal transducer activity	24	-1.80	0	0.02

MSigDB	Names of gene sets	Enriched in*	Category	# of genes	NES	NOM.p.val	FDR.q.val
	NEUROPEPTIDE_RECEPTOR_ACTIVITY			20	-2.14	0	0
	MONOVALENT_INORGANIC_CATION_ TRANSMEMBRANE_TRANSPORTER_ACTIVITY		transpoter activity	32	-2.03	0	0
	HYDROGEN_ION_TRANSMEMBRANE_ TRANSPORTER_ACTIVITY			26	-1.96	0	0
	ANION_CHANNEL_ACTIVITY			17	-1.97	0	0.01
	CHLORIDE_CHANNEL_ACTIVITY			16	-1.93	0	0.01
	SODIUM_CHANNEL_ACTIVITY			15	-1.89	0	0.01

Normal mucosal tissues (nm), colorectal tumors (crc)
